# Supplementary material for: Common metabolic constraints on dive duration in endothermic and ectothermic vertebrates
Source: PeerJ. 2016 Oct 12;4:e2569. doi: 10.7717/peerj.2569 (PMC5068442; doi:10.7717/peerj.2569)
Supplement: Appendix S1 — Body mass (M, in g), temperature (T, in °C), median dive time (median DT, in minutes), maximum dive time (max DT, in minutes), and the per species median and max. dive time data (in minutes) for amphibians, reptiles, birds and mammals. [file peerj-04-2569-s001.docx]

**Appendix 1**: Body mass (M, in g), temperature (T, in ^o^C), median dive time (median DT, in minutes), maximum dive time (max DT, in minutes), and the per species median and max. dive time data (in minutes) for amphibians, reptiles, birds and mammals. The numbers found in square parentheses in the last four columns refer to references, which are listed immediately below the appendix.

| **Species** | **M** | **T** | **Med. DT** | **Max. DT** | **Med. DT per species** | **Max. DT per species** | **M, ref.** | **T, ref.** | **Med DT, ref.** | **Max DT, ref.** |
| --- | --- | --- | --- | --- | --- | --- | --- | --- | --- | --- |
| AMPHIBIANS |  |  |  |  |  |  |  |  |  |  |
| *Triturus alpestris* | 2.6 | 10.0 | 5.9 |  |  |  | [1] | [1] | [1] |  |
| *Triturus alpestris* | 2.6 | 15.0 | 3.6 |  | *3.6* |  | [1] | [1] | [1] |  |
| *Triturus alpestris* | 2.6 | 20.0 | 2.3 |  |  |  | [1] | [1] | [1] |  |
| *Triturus alpestris* | 2.6 | 25.0 | 1.1 |  |  |  | [1] | [1] | [1] |  |
| REPTILES |  |  |  |  |  |  |  |  |  |  |
| *Crocodylus johnstoni* | 5060 | 23.5 |  | 344.5 |  |  | [2] | [2] |  | [2] |
| *Crocodylus johnstoni* | 5600 | 23.5 |  | 145.3 |  |  | [2] | [2] |  | [2] |
| *Crocodylus johnstoni* | 5920 | 23.5 |  | 186.2 |  |  | [2] | [2] |  | [2] |
| *Crocodylus johnstoni* | 6350 | 23.5 |  | 145.2 |  |  | [2] | [2] |  | [2] |
| *Crocodylus johnstoni* | 6500 | 23.5 |  | 237.4 |  |  | [2] | [2] |  | [2] |
| *Crocodylus johnstoni* | 9800 | 23.5 |  | 177.8 |  |  | [2] | [2] |  | [2] |
| *Crocodylus johnstoni* | 11560 | 23.5 |  | 152.7 |  |  | [2] | [2] |  | [2] |
| *Crocodylus johnstoni* | 13000 | 23.5 |  | 188.1 |  |  | [2] | [2] |  | [2] |
| *Crocodylus johnstoni* | 15460 | 23.5 |  | 133.1 |  |  | [2] | [2] |  | [2] |
| *Crocodylus johnstoni* | 21960 | 23.5 |  | 188.1 |  |  | [2] | [2] |  | [2] |
| *Crocodylus johnstoni* | 42490 | 23.5 |  | 401.3 |  | *401.3* | [2] | [2] |  | [2] |
| *Crocodylus johnstoni* | 6500 | 23.5 | 6.1 |  |  |  | [2] | [2] | [2] |  |
| *Crocodylus johnstoni* | 10500 | 23.5 | 6.3 |  |  |  | [2] | [2] | [2] |  |
| *Crocodylus johnstoni* | 28500 | 23.5 | 6.9 |  |  |  | [2] | [2] | [2] |  |
| *Crocodylus johnstoni* | 9880 | 21.6 | 33.4 |  |  |  | [3] | [3] | [3] |  |
| *Crocodylus johnstoni* | 9880 | 22.4 | 23.1 |  |  |  | [3] | [3] | [3] |  |
| *Crocodylus johnstoni* | 9880 | 23.4 | 22.0 |  |  |  | [3] | [3] | [3] |  |
| *Crocodylus johnstoni* | 9880 | 24.7 | 21.1 |  |  |  | [3] | [3] | [3] |  |
| *Crocodylus johnstoni* | 9880 | 26.5 | 17.1 |  |  |  | [3] | [3] | [3] |  |
| *Crocodylus johnstoni* | 9880 | 28.9 | 21.2 |  |  |  | [3] | [3] | [3] |  |
| *Crocodylus johnstoni* | 9880 | 31.9 | 14.7 |  |  |  | [3] | [3] | [3] |  |
| *Crocodylus johnstoni* | 5400 | 22.5 | 26.8 |  |  |  | [3] | [3] | [3] |  |
| *Crocodylus johnstoni* | 4000 | 22.5 | 20.2 |  | *20.2* |  | [3] | [3] | [3] |  |
| *Crocodylus johnstoni* | 16000 | 22.5 | 13.3 |  |  |  | [3] | [3] | [3] |  |
| *Crocodylus johnstoni* | 9200 | 22.5 | 30.3 |  |  |  | [3] | [3] | [3] |  |
| *Crocodylus johnstoni* | 14800 | 22.5 | 17.8 |  |  |  | [3] | [3] | [3] |  |
| *Crocodylus johnstoni* | 5400 | 22.5 |  | 94.7 |  |  | [3] | [3] |  | [3] |
| *Crocodylus johnstoni* | 4000 | 22.5 |  | 75.3 |  |  | [3] | [3] |  | [3] |
| *Crocodylus johnstoni* | 16000 | 22.5 |  | 67.9 |  |  | [3] | [3] |  | [3] |
| *Crocodylus johnstoni* | 9200 | 22.5 |  | 117.5 |  |  | [3] | [3] |  | [3] |
| *Crocodylus johnstoni* | 14800 | 22.5 |  | 119.6 |  |  | [3] | [3] |  | [3] |
| *Crocodylus porosus* | 9750 | 25.0 | 2.7 | 30.0 |  | *30.0* | [4] | [4] | [4] | [4] |
| *Crocodylus porosus* | 2250 | 25.0 | 3.1 |  | *3.1* |  | [5] | [5] | [5] |  |
| *Crocodylus porosus* | 2250 | 25.0 | 19.6 |  |  |  | [5] | [5] | [5] |  |
| *Amblyrhynchus cristatus* | 2254 | 23.1 | 3.1 | 6.3 | *3.1* |  | [6] | [6] | [6] | [6] |
| *Amblyrhynchus cristatus* | 1006 | 25.5 |  | 50.0 |  | *50.0* | [7] | [7] |  | [7] |
| *Eulamprus quoyii* | 19.7 | 19.1 | 7.4 |  | *7.4* |  | [8] | [8] | [8] |  |
| *Iguana iguana* | 895 | 25.5 | 82.0 | 270.0 | *82.0* | *270.0* | [8] | [8] | [8] | [8] |
| *Oligosoma smithi* | 4.2 | 17.0 | 2.5 |  | *2.5* |  | [8] | [8] | [8] |  |
| *Oligosoma smithi* | 4.8 | 17.0 |  | 6.6 |  |  | [8] | [8] |  | [8] |
| *Oligosoma smithi* | 13.2 | 17.3 | 5.0 | 20.5 | *5.0* | *20.5* | [8] | [8] | [8] | [8] |
| *Acalyptophis peronii* | 205 | 26.1 | 22.5 | 46.1 |  |  | [9] | [9] | [9] | [9] |
| *Acalyptophis peronii* | 285 | 26.1 | 13.1 | 27.4 |  |  | [9] | [9] | [9] | [9] |
| *Acalyptophis peronii* | 222 | 26.1 | 24.3 | 37.1 | *24.3* |  | [9] | [9] | [9] | [9] |
| *Acalyptophis peronii* | 248 | 26.1 | 46.6 | 54.1 |  | *54.1* | [9] | [9] | [9] | [9] |
| *Acrochordus arafurae* | 2550 | 26.0 | 11.1 | 56.9 |  |  | [10] | [10] | [10] | [10] |
| *Acrochordus arafurae* | 2320 | 26.0 | 5.5 | 53.7 |  |  | [10] | [10] | [10] | [10] |
| *Acrochordus arafurae* | 1920 | 26.0 | 7.2 | 55.4 |  |  | [10] | [10] | [10] | [10] |
| *Acrochordus arafurae* | 2000 | 26.0 | 5.7 | 153.7 | *5.7* | *153.7* | [10] | [10] | [10] | [10] |
| *Acrochordus arafurae* | 2000 | 26.0 | 3.8 | 56.4 |  |  | [10] | [10] | [10] | [10] |
| *Acrochordus granulatus* | 326 | 26.1 | 40.6 | 116.9 |  | *116.9* | [9] | [9] | [9] | [9] |
| *Acrochordus granulatus* | 326 | 30.0 | 3.2 |  | *3.2* |  | [11] | [11] | [11] |  |
| *Aipysurus duboisi* | 355 | 25.0 | 22.8 |  |  |  | [11] | [11] | [11] |  |
| *Aipysurus duboisi* | 355 | 28.0 | 16.7 |  | *16.7* |  | [11] | [11] | [11] |  |
| *Aipysurus duboisi* | 355 | 26.1 | 16.3 | 45.8 |  | *45.8* | [9] | [9] | [9] | [9] |
| *Aipysurus laevis* | 533 | 23.0 | 20.0 | 30.0 | *20.0* | *30.0* | [11] | [11] | [11] | [11] |
| *Aipysurus laevis* | 533 | 26.0 | 7.1 | 8.6 |  |  | [11] | [11] | [11] | [11] |
| *Emydocephalus annulatus* | 141 | 26.0 | 14.3 | 17.3 | *14.3* | *17.3* | [11] | [11] | [11] | [11] |
| *Hydrophis belcheri* | 201 | 26.1 | 7.3 | 31.2 |  | *31.2* | [9] | [9] | [9] | [9] |
| *Hydrophis belcheri* | 227 | 26.1 | 15.4 |  |  |  | [9] | [9] | [9] |  |
| *Hydrophis belcheri* | 464 | 26.1 | 11.0 | 25.4 | *11.0* |  | [9] | [9] | [9] | [9] |
| *Hydrophis elegans* | 388 | 26.1 | 4.4 | 13.2 | *4.4* | *13.2* | [9] | [9] | [9] | [9] |
| *Lapemis hardwickii* | 420 | 26.1 | 7.6 | 14.2 | *7.6* | *14.2* | [9] | [9] | [9] | [9] |
| *Lapemis hardwickii* | 102.2 | 17.0 | 4.2 | 10.6 |  |  | [11] | [11] | [11] | [11] |
| *Laticauda saintgironsi* | 390 | 26.3 | 17.0 | 138.0 | *17.0* | *138.0* | [12] | [12] | [12] | [12] |
| *Pelamis platurus* | 118 | 20.0 | 52.8 | 106.8 |  |  | [13] | [13] | [13] | [13] |
| *Pelamis platurus* | 147 | 20.0 | 28.4 | 55.6 |  |  | [13] | [13] | [13] | [13] |
| *Pelamis platurus* | 160 | 20.0 | 48.0 | 90.5 |  |  | [13] | [13] | [13] | [13] |
| *Pelamis platurus* | 161 | 20.0 | 24.9 | 32.3 |  |  | [13] | [13] | [13] | [13] |
| *Pelamis platurus* | 161 | 20.0 | 28.9 | 86.1 |  |  | [13] | [13] | [13] | [13] |
| *Pelamis platurus* | 101 | 20.0 | 18.5 | 27.2 |  |  | [13] | [13] | [13] | [13] |
| *Pelamis platurus* | 120 | 22.5 | 11.2 | 14.0 |  |  | [13] | [13] | [13] | [13] |
| *Pelamis platurus* | 90 | 22.5 | 43.7 | 72.0 |  |  | [13] | [13] | [13] | [13] |
| *Pelamis platurus* | 110 | 22.5 | 42.4 | 137.4 |  |  | [13] | [13] | [13] | [13] |
| *Pelamis platurus* | 150 | 22.5 | 21.0 | 68.2 |  |  | [13] | [13] | [13] | [13] |
| *Pelamis platurus* | 195 | 22.5 | 19.4 | 43.7 |  |  | [13] | [13] | [13] | [13] |
| *Pelamis platurus* | 130 | 20.0 | 54.5 | 139.3 |  | *139.3* | [13] | [13] | [13] | [13] |
| *Pelamis platurus* | 128 | 20.0 | 74.6 | 102.5 |  |  | [13] | [13] | [13] | [13] |
| *Pelamis platurus* | 150 | 20.0 | 37.7 | 103.0 | *37.7* |  | [13] | [13] | [13] | [13] |
| *Pelamis platurus* | 140 | 22.5 | 50.3 | 213.0 |  |  | [13] | [13] | [13] | [13] |
| *Apalone ferox* | 1123.3 | 25.0 | 5.4 |  |  |  | [14] | [14] | [14] |  |
| *Apalone ferox* | 1123.3 | 15.0 | 18.6 |  | *18.6* |  | [14] | [14] | [14] |  |
| *Caretta caretta* | 72500 | 27.4 | 25.0 |  |  |  | [15] | [15] | [15] |  |
| *Caretta caretta* | 72500 | 26.6 | 15.0 |  |  |  | [15] | [15] | [15] |  |
| *Caretta caretta* | 72500 | 26.3 | 12.0 |  |  |  | [15] | [15] | [15] |  |
| *Caretta caretta* | 72500 | 26.5 | 20.1 |  |  |  | [15] | [15] | [15] |  |
| *Caretta caretta* | 72500 | 27.3 | 20.0 |  |  |  | [15] | [15] | [15] |  |
| *Caretta caretta* | 72500 | 28.7 | 19.8 |  |  |  | [15] | [15] | [15] |  |
| *Caretta caretta* | 72500 | 26.5 | 15.0 |  |  |  | [15] | [15] | [15] |  |
| *Caretta caretta* | 72500 | 23.5 | 25.0 |  | *25.0* |  | [15] | [15] | [15] |  |
| *Caretta caretta* | 72500 | 24.2 | 36.0 |  |  |  | [15] | [15] | [15] |  |
| *Caretta caretta* | 72500 | 23.1 | 34.0 |  |  |  | [15] | [15] | [15] |  |
| *Caretta caretta* | 72500 | 25.1 | 45.0 |  |  |  | [15] | [15] | [15] |  |
| *Caretta caretta* | 72500 | 25.9 | 22.0 |  |  |  | [15] | [15] | [15] |  |
| *Caretta caretta* | 72500 | 25.2 | 25.0 |  |  |  | [15] | [15] | [15] |  |
| *Caretta caretta* | 72500 | 23.0 | 58.0 |  |  |  | [15] | [15] | [15] |  |
| *Caretta caretta* | 72500 | 22.3 | 29.0 |  |  |  | [15] | [15] | [15] |  |
| *Caretta caretta* | 72500 | 21.2 | 50.0 |  |  |  | [15] | [15] | [15] |  |
| *Caretta caretta* | 72500 | 22.2 | 38.0 |  |  |  | [15] | [15] | [15] |  |
| *Caretta caretta* | 72500 | 20.4 | 66.0 |  |  |  | [15] | [15] | [15] |  |
| *Caretta caretta* | 72500 | 20.3 | 58.0 |  |  |  | [15] | [15] | [15] |  |
| *Caretta caretta* | 72500 | 20.0 | 57.0 |  |  |  | [15] | [15] | [15] |  |
| *Caretta caretta* | 91500 | 27.4 | 17.0 |  |  |  | [15] | [15] | [15] |  |
| *Caretta caretta* | 91500 | 24.2 | 18.0 |  |  |  | [15] | [15] | [15] |  |
| *Caretta caretta* | 91500 | 28.2 | 15.1 |  |  |  | [15] | [15] | [15] |  |
| *Caretta caretta* | 91500 | 28.8 | 14.0 |  |  |  | [15] | [15] | [15] |  |
| *Caretta caretta* | 91500 | 27.8 | 17.5 |  |  |  | [15] | [15] | [15] |  |
| *Caretta caretta* | 91500 | 26.5 | 18.0 |  |  |  | [15] | [15] | [15] |  |
| *Caretta caretta* | 91500 | 26.7 | 19.0 |  |  |  | [15] | [15] | [15] |  |
| *Caretta caretta* | 91500 | 25.2 | 24.0 |  |  |  | [15] | [15] | [15] |  |
| *Caretta caretta* | 91500 | 24.6 | 26.0 |  |  |  | [15] | [15] | [15] |  |
| *Caretta caretta* | 91500 | 23.8 | 30.0 |  |  |  | [15] | [15] | [15] |  |
| *Caretta caretta* | 91500 | 23.7 | 36.0 |  |  |  | [15] | [15] | [15] |  |
| *Caretta caretta* | 91500 | 23.1 | 36.5 |  |  |  | [15] | [15] | [15] |  |
| *Caretta caretta* | 91500 | 22.0 | 37.0 |  |  |  | [15] | [15] | [15] |  |
| *Caretta caretta* | 52000 | 25.9 | 5.5 | 52.7 |  |  | [16] | [16] | [16] | [16] |
| *Caretta caretta* | 52000 | 25.7 |  | 105.5 |  |  | [16] | [16] |  | [16] |
| *Caretta caretta* | 52000 | 25.0 |  | 62.6 |  |  | [16] | [16] |  | [16] |
| *Caretta caretta* | 52000 | 22.6 |  | 125.3 |  |  | [16] | [16] |  | [16] |
| *Caretta caretta* | 52000 | 20.0 |  | 150.0 |  |  | [16] | [16] |  | [16] |
| *Caretta caretta* | 52000 | 17.1 |  | 253.8 |  |  | [16] | [16] |  | [16] |
| *Caretta caretta* | 52000 | 16.4 |  | 248.9 |  |  | [16] | [16] |  | [16] |
| *Caretta caretta* | 52000 | 14.8 | 341.0 | 410.4 |  |  | [16] | [16] | [16] | [16] |
| *Caretta caretta* | 34500 | 13.5 |  | 360.6 |  |  | [17] | [17] |  | [17] |
| *Caretta caretta* | 34500 | 15.1 |  | 269.0 |  |  | [17] | [17] |  | [17] |
| *Caretta caretta* | 34500 | 15.5 |  | 198.6 |  |  | [17] | [17] |  | [17] |
| *Caretta caretta* | 34500 | 17.6 |  | 159.2 |  |  | [17] | [17] |  | [17] |
| *Caretta caretta* | 34500 | 20.2 |  | 118.3 |  |  | [17] | [17] |  | [17] |
| *Caretta caretta* | 34500 | 16.4 | 120.0 |  |  |  | [17] | [17] | [17] |  |
| *Caretta caretta* | 42130 | 13.4 |  | 360.6 |  |  | [17] | [17] |  | [17] |
| *Caretta caretta* | 42130 | 13.7 |  | 269.0 |  |  | [17] | [17] |  | [17] |
| *Caretta caretta* | 42130 | 14.3 |  | 221.1 |  |  | [17] | [17] |  | [17] |
| *Caretta caretta* | 42130 | 14.5 |  | 270.4 |  |  | [17] | [17] |  | [17] |
| *Caretta caretta* | 42130 | 14.8 |  | 121.1 |  |  | [17] | [17] |  | [17] |
| *Caretta caretta* | 42130 | 15.0 |  | 301.4 |  |  | [17] | [17] |  | [17] |
| *Caretta caretta* | 42130 | 15.3 |  | 160.6 |  |  | [17] | [17] |  | [17] |
| *Caretta caretta* | 42130 | 15.6 |  | 331.0 |  |  | [17] | [17] |  | [17] |
| *Caretta caretta* | 42130 | 16.1 |  | 269.0 |  |  | [17] | [17] |  | [17] |
| *Caretta caretta* | 42130 | 16.9 |  | 181.7 |  |  | [17] | [17] |  | [17] |
| *Caretta caretta* | 42130 | 20.7 |  | 109.9 |  |  | [17] | [17] |  | [17] |
| *Caretta caretta* | 42130 | 21.6 |  | 105.6 |  |  | [17] | [17] |  | [17] |
| *Caretta caretta* | 42130 | 21.9 |  | 119.7 |  |  | [17] | [17] |  | [17] |
| *Caretta caretta* | 42130 | 22.0 |  | 97.2 |  |  | [17] | [17] |  | [17] |
| *Caretta caretta* | 42130 | 22.3 |  | 78.9 |  |  | [17] | [17] |  | [17] |
| *Caretta caretta* | 42130 | 22.7 |  | 59.2 |  |  | [17] | [17] |  | [17] |
| *Caretta caretta* | 42130 | 17.6 | 26.0 |  |  |  | [17] | [17] | [17] |  |
| *Caretta caretta* | 19650 | 12.5 |  | 480.3 |  | *480.3* | [17] | [17] |  | [17] |
| *Caretta caretta* | 19650 | 12.8 |  | 331.0 |  |  | [17] | [17] |  | [17] |
| *Caretta caretta* | 19650 | 12.9 |  | 362.0 |  |  | [17] | [17] |  | [17] |
| *Caretta caretta* | 19650 | 13.0 |  | 269.0 |  |  | [17] | [17] |  | [17] |
| *Caretta caretta* | 19650 | 13.4 |  | 239.4 |  |  | [17] | [17] |  | [17] |
| *Caretta caretta* | 19650 | 13.8 |  | 200.0 |  |  | [17] | [17] |  | [17] |
| *Caretta caretta* | 19650 | 15.1 |  | 242.3 |  |  | [17] | [17] |  | [17] |
| *Caretta caretta* | 19650 | 15.1 |  | 171.8 |  |  | [17] | [17] |  | [17] |
| *Caretta caretta* | 19650 | 15.9 |  | 95.8 |  |  | [17] | [17] |  | [17] |
| *Caretta caretta* | 19650 | 17.6 |  | 129.6 |  |  | [17] | [17] |  | [17] |
| *Caretta caretta* | 19650 | 19.1 |  | 116.9 |  |  | [17] | [17] |  | [17] |
| *Caretta caretta* | 19650 | 19.2 |  | 129.6 |  |  | [17] | [17] |  | [17] |
| *Caretta caretta* | 19650 | 19.2 |  | 98.6 |  |  | [17] | [17] |  | [17] |
| *Caretta caretta* | 19650 | 19.3 |  | 59.2 |  |  | [17] | [17] |  | [17] |
| *Caretta caretta* | 19650 | 20.4 |  | 63.4 |  |  | [17] | [17] |  | [17] |
| *Caretta caretta* | 19650 | 20.9 |  | 73.2 |  |  | [17] | [17] |  | [17] |
| *Caretta caretta* | 19650 | 21.1 |  | 36.6 |  |  | [17] | [17] |  | [17] |
| *Caretta caretta* | 19650 | 21.8 |  | 53.5 |  |  | [17] | [17] |  | [17] |
| *Caretta caretta* | 19650 | 22.3 |  | 22.5 |  |  | [17] | [17] |  | [17] |
| *Caretta caretta* | 19650 | 22.6 |  | 43.7 |  |  | [17] | [17] |  | [17] |
| *Caretta caretta* | 19650 | 17.4 | 115.0 |  |  |  | [17] | [17] | [17] |  |
| *Caretta caretta* | 50250 | 12.0 |  | 140.8 |  |  | [17] | [17] |  | [17] |
| *Caretta caretta* | 50250 | 13.2 |  | 119.7 |  |  | [17] | [17] |  | [17] |
| *Caretta caretta* | 50250 | 15.2 |  | 330.0 |  |  | [17] | [17] |  | [17] |
| *Caretta caretta* | 50250 | 22.5 |  | 32.4 |  |  | [17] | [17] |  | [17] |
| *Caretta caretta* | 50250 | 22.5 |  | 11.3 |  |  | [17] | [17] |  | [17] |
| *Caretta caretta* | 50250 | 23.4 |  | 57.7 |  |  | [17] | [17] |  | [17] |
| *Caretta caretta* | 50250 | 23.6 |  | 119.7 |  |  | [17] | [17] |  | [17] |
| *Caretta caretta* | 50250 | 24.2 |  | 46.5 |  |  | [17] | [17] |  | [17] |
| *Caretta caretta* | 50250 | 24.8 |  | 64.8 |  |  | [17] | [17] |  | [17] |
| *Caretta caretta* | 50250 | 25.5 |  | 105.6 |  |  | [17] | [17] |  | [17] |
| *Caretta caretta* | 50250 | 25.9 |  | 71.8 |  |  | [17] | [17] |  | [17] |
| *Caretta caretta* | 50250 | 26.3 |  | 56.3 |  |  | [17] | [17] |  | [17] |
| *Caretta caretta* | 50250 | 26.4 |  | 80.3 |  |  | [17] | [17] |  | [17] |
| *Caretta caretta* | 50250 | 27.1 |  | 42.3 |  |  | [17] | [17] |  | [17] |
| *Caretta caretta* | 50250 | 30.3 |  | 53.5 |  |  | [17] | [17] |  | [17] |
| *Caretta caretta* | 50250 | 30.7 |  | 70.4 |  |  | [17] | [17] |  | [17] |
| *Caretta caretta* | 50250 | 30.9 |  | 21.1 |  |  | [17] | [17] |  | [17] |
| *Caretta caretta* | 50250 | 31.0 |  | 62.0 |  |  | [17] | [17] |  | [17] |
| *Caretta caretta* | 50250 | 31.8 |  | 94.4 |  |  | [17] | [17] |  | [17] |
| *Caretta caretta* | 50250 | 32.6 |  | 77.5 |  |  | [17] | [17] |  | [17] |
| *Caretta caretta* | 55000 | 27.1 |  | 52.9 |  |  | [18] | [18] |  | [18] |
| *Caretta caretta* | 55000 | 27.1 |  | 39.7 |  |  | [18] | [18] |  | [18] |
| *Caretta caretta* | 55000 | 27.1 | 29.4 |  |  |  | [18] | [18] | [18] |  |
| *Caretta caretta* | 55000 | 27.1 | 5.9 |  |  |  | [18] | [18] | [18] |  |
| *Caretta caretta* | 74000 | 23.8 | 25.9 | 50.0 |  |  | [19] | [19] | [19] | [19] |
| *Caretta caretta* | 73000 | 23.8 | 39.9 | 70.0 |  |  | [19] | [19] | [19] | [19] |
| *Caretta caretta* | 82000 | 23.8 | 14.1 | 50.0 |  |  | [19] | [19] | [19] | [19] |
| *Caretta caretta* | 80000 | 23.8 | 35.4 | 60.0 |  |  | [19] | [19] | [19] | [19] |
| *Caretta caretta* | 75000 | 23.8 | 20.1 | 36.7 |  |  | [19] | [19] | [19] | [19] |
| *Caretta caretta* | 62500 | 23.8 | 21.3 | 56.7 |  |  | [19] | [19] | [19] | [19] |
| *Chelonia mydas* | 115200 | 26.9 | 15.0 | 25.0 |  |  | [20] | [20] | [20] | [20] |
| *Chelonia mydas* | 200000 | 28.4 | 24.6 | 45.8 |  |  | [20] | [20] | [20] | [20] |
| *Chelonia mydas* | 125200 | 28.4 | 19.4 | 31.1 |  |  | [20] | [20] | [20] | [20] |
| *Chelonia mydas* | 211000 | 27.7 | 18.1 | 30.9 |  |  | [20] | [20] | [20] | [20] |
| *Chelonia mydas* | 105600 | 28.0 | 28.6 | 51.0 |  |  | [21] | [21] | [21] | [21] |
| *Chelonia mydas* | 236500 | 28.0 | 36.0 | 55.0 |  |  | [21] | [21] | [21] | [21] |
| *Chelonia mydas* | 115000 | 26.0 | 38.8 |  |  |  | [22] | [22] | [22] |  |
| *Chelonia mydas* | 21600 | 26.2 | 14.3 |  |  |  | [23] | [23] | [23] |  |
| *Chelonia mydas* | 15000 | 27.9 | 11.9 |  |  |  | [23] | [23] | [23] |  |
| *Chelonia mydas* | 15600 | 25.3 | 15.2 |  |  |  | [23] | [23] | [23] |  |
| *Chelonia mydas* | 11700 | 25.6 | 12.6 |  |  |  | [23] | [23] | [23] |  |
| *Chelonia mydas* | 11600 | 26.6 | 12.8 |  |  |  | [23] | [23] | [23] |  |
| *Chelonia mydas* | 16900 | 25.8 | 11.9 |  |  |  | [23] | [23] | [23] |  |
| *Chelonia mydas* | 23600 | 21.9 | 22.7 |  | *22.7* |  | [23] | [23] | [23] |  |
| *Chelonia mydas* | 17100 | 21.7 | 25.9 |  |  |  | [23] | [23] | [23] |  |
| *Chelonia mydas* | 11100 | 26.6 | 12.8 |  |  |  | [24] | [24] | [24] |  |
| *Chelonia mydas* | 11600 | 25.6 | 12.6 |  |  |  | [24] | [24] | [24] |  |
| *Chelonia mydas* | 15700 | 25.3 | 15.2 |  |  |  | [24] | [24] | [24] |  |
| *Chelonia mydas* | 23800 | 21.9 | 22.7 |  |  |  | [24] | [24] | [24] |  |
| *Chelonia mydas* | 17200 | 21.7 | 25.9 |  |  |  | [24] | [24] | [24] |  |
| *Chelonia mydas* | 16300 | 25.8 | 11.9 |  |  |  | [24] | [24] | [24] |  |
| *Chrysemys dorbignyi* | 1740 | 27.0 | 7.7 | 31.6 | *7.7* | *31.6* | [25] | [25] | [25] | [25] |
| *Chrysemys dorbignyi* | 1755 | 18.0 | 14.1 | 35.0 |  |  | [25] | [25] | [25] | [25] |
| *Chrysemys picta* | 295.4 | 25.0 | 3.9 |  | *3.9* |  | [14] | [14] | [14] |  |
| *Chrysemys picta* | 295.4 | 15.0 | 5.7 |  |  |  | [14] | [14] | [14] |  |
| *Emydura macquarii* | 1900 | 25.9 | 9.6 | 85.2 |  |  | [26] | [26] | [26] | [26] |
| *Emydura macquarii* | 1595 | 15.0 | 31.7 | 166.0 |  | *166.0* | [27] | [27] | [27] | [27] |
| *Emydura macquarii* | 1600 | 23.0 | 6.6 |  |  |  | [27] | [27] | [27] |  |
| *Emydura macquarii* | 1600 | 30.0 | 7.5 |  | *7.5* |  | [27] | [27] | [27] |  |
| *Eretmochelys imbricata* | 1737.4 | 27.0 | 16.5 |  |  |  | [28] | [28] | [28] |  |
| *Eretmochelys imbricata* | 1817.4 | 27.0 | 11.6 |  |  |  | [28] | [28] | [28] |  |
| *Eretmochelys imbricata* | 2144 | 27.0 | 15.0 |  |  |  | [28] | [28] | [28] |  |
| *Eretmochelys imbricata* | 2962.8 | 27.0 | 13.2 |  |  |  | [28] | [28] | [28] |  |
| *Eretmochelys imbricata* | 3595.4 | 27.0 | 14.0 |  |  |  | [28] | [28] | [28] |  |
| *Eretmochelys imbricata* | 5393.9 | 27.0 | 23.8 |  |  |  | [28] | [28] | [28] |  |
| *Eretmochelys imbricata* | 5552.4 | 27.0 | 15.7 |  |  |  | [28] | [28] | [28] |  |
| *Eretmochelys imbricata* | 5628.8 | 27.0 | 20.7 |  | *20.7* |  | [28] | [28] | [28] |  |
| *Eretmochelys imbricata* | 5732 | 27.0 | 16.7 |  |  |  | [28] | [28] | [28] |  |
| *Eretmochelys imbricata* | 5773.3 | 27.0 | 17.4 |  |  |  | [28] | [28] | [28] |  |
| *Eretmochelys imbricata* | 6088.9 | 27.0 | 13.1 |  |  |  | [28] | [28] | [28] |  |
| *Eretmochelys imbricata* | 7564.6 | 27.0 | 20.9 |  |  |  | [28] | [28] | [28] |  |
| *Eretmochelys imbricata* | 8124.3 | 27.0 | 13.6 |  |  |  | [28] | [28] | [28] |  |
| *Eretmochelys imbricata* | 8464 | 27.0 | 11.7 |  |  |  | [28] | [28] | [28] |  |
| *Eretmochelys imbricata* | 8559.8 | 27.0 | 24.7 |  |  |  | [28] | [28] | [28] |  |
| *Eretmochelys imbricata* | 9031.7 | 27.0 | 34.6 |  |  |  | [28] | [28] | [28] |  |
| *Eretmochelys imbricata* | 10716 | 27.0 | 33.3 |  |  |  | [28] | [28] | [28] |  |
| *Eretmochelys imbricata* | 12024 | 27.0 | 25.4 |  |  |  | [28] | [28] | [28] |  |
| *Eretmochelys imbricata* | 16286 | 27.0 | 44.6 |  |  |  | [28] | [28] | [28] |  |
| *Eretmochelys imbricata* | 3700 | 26.0 | 21.0 | 57.7 |  |  | [29] | [29] | [29] | [29] |
| *Eretmochelys imbricata* | 3800 | 26.0 | 22.9 | 81.1 |  | *81.1* | [29] | [29] | [29] | [29] |
| *Eretmochelys imbricata* | 16200 | 26.0 | 24.0 | 77.5 |  |  | [29] | [29] | [29] | [29] |
| *Eretmochelys imbricata* | 17200 | 26.0 | 21.5 | 52.0 |  |  | [29] | [29] | [29] | [29] |
| *Lepidochelys kempii* | 7400 | 22.0 | 34.7 | 147.7 | *34.7* |  | [30] | [30] | [30] | [30] |
| *Lepidochelys kempii* | 9800 | 22.0 | 45.5 | 115.3 |  |  | [30] | [30] | [30] | [30] |
| *Lepidochelys kempii* | 27940 | 22.6 | 87.5 |  |  |  | [30] | [30] | [30] |  |
| *Lepidochelys kempii* | 27941 | 21.1 | 76.0 |  |  |  | [30] | [30] | [30] |  |
| *Lepidochelys kempii* | 27940 | 23.3 | 28.7 |  |  |  | [30] | [30] | [30] |  |
| *Lepidochelys olivacea* | 39000 | 31.6 | 49.8 |  |  |  | [31] | [31] | [31] |  |
| *Lepidochelys olivacea* | 39000 | 26.9 | 35.5 |  |  |  | [31] | [31] | [31] |  |
| *Lepidochelys olivacea* | 32600 | 24.3 | 37.2 |  | *37.2* |  | [32] | [32] | [32] |  |
| *Lepidochelys olivacea* | 31800 | 27.1 | 48.0 |  |  |  | [32] | [32] | [32] |  |
| *Lepidochelys olivacea* | 34300 | 26.4 | 33.7 |  |  |  | [32] | [32] | [32] |  |
| *Lepidochelys olivacea* | 33100 | 27.2 | 24.5 |  |  |  | [32] | [32] | [32] |  |
| *Rheodytes leukops* | 1325 | 15.0 | 98.6 | 538.0 |  |  | [27] | [27] | [27] | [27] |
| *Rheodytes leukops* | 1325 | 23.0 | 36.1 |  |  |  | [27] | [27] | [27] |  |
| *Rheodytes leukops* | 1325 | 30.0 | 15.2 |  |  |  | [27] | [27] | [27] |  |
|  |  |  |  |  |  |  |  |  |  |  |
| BIRDS |  |  |  |  |  |  |  |  |  |  |
| *Aechmophorus occidentalis* | 900.0 | 38.5 | 0.5 | 1.2 | *0.5* | *1.2* | [33] | [34] | [33] | [33] |
| *Alca torda* | 730.6 | 41.3 | 0.8 |  |  |  | [33] | [34] | [35] |  |
| *Alca torda* | 616.0 | 41.3 | 0.6 |  | *0.6* |  | [36] | [34] | [36] |  |
| *Alle alle* | 149.0 | 41.5^[[1]](#endnote-1)^ | 0.9 | 1.5 | *0.9* | *1.5* | [37] | [34] | [37] | [37] |
| *Anhinga melanogaster* | 1400 | 40.9^[[2]](#endnote-2)^ | 0.6 | 1.8 | *0.6* | *1.8* | [38] | [34] | [38] | [38] |
| *Aptenodytes forsteri* | 29500 | 36.1 | 5.5 | 10.4 |  |  | [39] | [34] | [39] | [39] |
| *Aptenodytes forsteri* | 27000 | 36.1 | 5.6 | 9.1 |  |  | [39] | [34] | [39] | [39] |
| *Aptenodytes forsteri* | 26800 | 36.1 | 6.0 | 7.4 |  |  | [39] | [34] | [39] | [39] |
| *Aptenodytes forsteri* | 26560 | 36.1 |  | 15.8 |  |  | [40] | [34] |  | [40] |
| *Aptenodytes forsteri* | 25500 | 36.1 | 6.0 | 12.0 |  |  | [41] | [34] | [41] | [41] |
| *Aptenodytes forsteri* | 25400 | 36.1 | 3.7 | 5.0 |  |  | [42] | [34] | [42] | [42] |
| *Aptenodytes forsteri* | 25300 | 36.1 | 3.2 | 15.5 |  |  | [43] | [34] | [43] | [43] |
| *Aptenodytes forsteri* | 25000 | 36.1 | 7.0 | 9.6 |  |  | [39] | [34] | [39] | [39] |
| *Aptenodytes forsteri* | 24800 | 36.1 | 4.4 | 4.6 | *4.4* |  | [39] | [34] | [39] | [39] |
| *Aptenodytes forsteri* | 24700 | 36.1 | 3.6 | 4.6 |  |  | [39] | [34] | [39] | [39] |
| *Aptenodytes forsteri* | 24500 | 36.1 | 2.4 | 18.0 |  |  | [44] | [34] | [44] | [44] |
| *Aptenodytes forsteri* | 24300 | 36.1 | 3.4 | 5.3 |  |  | [39] | [34] | [39] | [39] |
| *Aptenodytes forsteri* | 24200 | 36.1 | 5.2 | 14.8 |  |  | [45] | [34] | [45] | [45] |
| *Aptenodytes forsteri* | 23600 | 36.1 | 4.1 | 21.3 |  | *21.3* | [43] | [34] | [43] | [43] |
| *Aptenodytes forsteri* | 25511 | 36.1 | 3.3 | 16.1 |  |  | [46] | [34] | [46] | [46] |
| *Aptenodytes patagonicus* | 12860 | 36.1ii | 5.5 | 7.7 |  |  | [47] | [34] | [47] | [47] |
| *Aptenodytes patagonicus* | 12000 | 36.1ii |  | 8.7 |  |  | [48] | [34] |  | [48] |
| *Aptenodytes patagonicus* | 12000 | 36.1ii |  | 8.4 |  |  | [48] | [34] |  | [48] |
| *Aptenodytes patagonicus* | 10900 | 36.1ii | 4.1 | 6.3 |  |  | [49] | [34] | [49] | [49] |
| *Aptenodytes patagonicus* | 10900 | 36.1ii | 2.5 | 9.2 |  | *9.2* | [50] | [34] | [50] | [50] |
| *Aptenodytes patagonicus* | 10600 | 36.1ii | 2.4 | 7.6 |  |  | [51] | [34] | [51] | [51] |
| *Aptenodytes patagonicus* | 11222 | 36.1ii | 2.6 | 8.0 | *2.6* |  | [46] | [34] | [46] | [46] |
| *Aythya australis* | 900 | 42.3ii | 0.1 |  | *0.1* |  | [52] | [34] | [52] |  |
| *Aythya ferina* | 1000 | 42.3ii | 0.2 | 0.3 | *0.2* | *0.3* | [33] | [34] | [53] | [53] |
| *Aythya fuligula* | 607 | 42.3ii | 0.3 | 0.6 |  | *0.6* | [54] | [34] | [54] | [54] |
| *Aythya fuligula* | 653.5 | 42.3ii | 0.2 | 0.4 | *0.2* |  | [53] | [34] | [53] | [53] |
| *Aythya marila* | 1100 | 42.3ii | 0.3 | 0.5 | *0.3* | *0.5* | [33] | [34] | [33] | [33] |
| *Aythya novaeseelandiae* | 700 | 42.3ii | 0.3 |  | *0.3* |  | [33] | [34] | [52] |  |
| *Aythya valisineria* | 1238 | 42.3ii |  | 0.4 |  | *0.4* | [55] | [34] |  | [55] |
| *Biziura lobata* | 2000 | 41.5i | 0.3 |  | *0.3* |  | [52] | [34] | [52] |  |
| *Brachyramphus marmoratus* | 217 | 41.5i | 0.4 |  |  |  | [56] | [34] | [56] |  |
| *Brachyramphus marmoratus* | 216 | 41.5i | 0.4 | 0.8 | *0.4* |  | [57] | [34] | [57] | [57] |
| *Bucephala clangula* | 1000 | 41.5i | 0.2 | 0.3 | *0.2* | *0.3* | [33] | [34] | [58] | [58] |
| *Bucephala islandica* | 1000 | 41.5ii | 0.4 | 0.6 | *0.4* | *0.6* | [33] | [34] | [59] | [59] |
| *Cepphus grylle* | 400 | 40.4 | 1.1 | 2.5 | *1.1* | *2.5* | [33] | [34] | [33] | [33] |
| *Cerorhinca monocerata* | 612 | 41.5i | 1.1 |  |  |  | [60] | [34] | [60] |  |
| *Cerorhinca monocerata* | 592 | 41.5i | 0.8 |  |  |  | [60] | [34] | [60] |  |
| *Cerorhinca monocerata* | 591 | 41.5i | 0.8 |  |  |  | [60] | [34] | [60] |  |
| *Cerorhinca monocerata* | 580 | 41.5i | 1.0 |  |  |  | [60] | [34] | [60] |  |
| *Cerorhinca monocerata* | 565 | 41.5i | 1.0 |  |  |  | [60] | [34] | [60] |  |
| *Cerorhinca monocerata* | 526 | 41.5i | 1.0 |  |  |  | [60] | [34] | [60] |  |
| *Cerorhinca monocerata* | 520 | 41.5i | 0.7 |  |  |  | [60] | [34] | [60] |  |
| *Cerorhinca monocerata* | 515 | 41.5i | 0.8 |  | *0.8* |  | [60] | [34] | [60] |  |
| *Cerorhinca monocerata* | 557.9 | 41.5i | 0.7 | 2.2 |  | *2.2* | [46] | [34] | [46] | [46] |
| *Cerorhinca monocerata* | 520 | 41.5i | 0.8 | 1.2 |  |  | [61] | [34] | [61] | [61] |
| *Clangula hyemalis* | 700 | 41.5i | 0.5 | 0.8 | *0.5* |  | [62] | [34] | [63] | [63] |
| *Clangula hyemalis* | 900 | 41.5i | 0.7 | 0.9 |  | *0.9* | [33] | [34] | [33] | [33] |
| *Diomedea cauta* | 3900 | 39.8ii | 0.1 | 0.3 | *0.1* | *0.3* | [33] | [34] | [64] | [64] |
| *Diomedea chrysostoma* | 3500 | 39.8ii | 0.1 | 0.2 | *0.1* | *0.2* | [33] | [34] | [33] | [33] |
| *Diomedea melanophrys* | 4000 | 39.8ii | 0.1 | 0.2 | *0.1* | *0.2* | [33] | [34] | [65] | [65] |
| *Eudyptes chrysocome* | 2500 | 41.5i | 1.3 | 4.2 | *1.3* | *4.2* | [46] | [34] | [46] | [46] |
| *Eudyptes chrysolophus* | 3500 | 41.5i | 1.5 | 6.3 |  | *6.3* | [46] | [34] | [46] | [46] |
| *Eudyptes chrysolophus* | 3600 | 41.5i | 1.7 | 1.8 |  |  | [66] | [34] | [66] | [66] |
| *Eudyptes chrysolophus* | 3300 | 41.5i | 1.4 | 3.7 |  |  | [66] | [34] | [66] | [66] |
| *Eudyptes chrysolophus* | 3100 | 41.5i | 1.6 | 2.7 |  |  | [66] | [34] | [66] | [66] |
| *Eudyptes chrysolophus* | 3900 | 41.5i | 1.5 | 2.8 |  |  | [66] | [34] | [66] | [66] |
| *Eudyptes chrysolophus* | 3600 | 41.5i | 1.4 | 2.4 |  |  | [66] | [34] | [66] | [66] |
| *Eudyptes chrysolophus* | 3300 | 41.5i | 1.4 | 3.0 |  |  | [66] | [34] | [66] | [66] |
| *Eudyptes chrysolophus* | 4000 | 41.5i | 1.6 | 3.1 |  |  | [66] | [34] | [66] | [66] |
| *Eudyptes chrysolophus* | 3400 | 41.5i | 1.4 | 2.6 |  |  | [66] | [34] | [66] | [66] |
| *Eudyptes chrysolophus* | 3800 | 41.5i | 1.1 | 3.0 |  |  | [66] | [34] | [66] | [66] |
| *Eudyptes chrysolophus* | 3800 | 41.5i | 1.3 | 2.9 |  |  | [66] | [34] | [66] | [66] |
| *Eudyptes chrysolophus* | 4100 | 41.5i | 1.6 | 3.9 |  |  | [66] | [34] | [66] | [66] |
| *Eudyptes chrysolophus* | 3600 | 41.5i | 1.3 | 2.9 |  |  | [66] | [34] | [66] | [66] |
| *Eudyptes chrysolophus* | 3400 | 41.5i | 1.5 | 2.9 | *1.5* |  | [66] | [34] | [66] | [66] |
| *Eudyptes chrysolophus* | 3400 | 41.5i | 1.4 | 2.6 |  |  | [67] | [34] | [67] | [67] |
| *Eudyptes chrysolophus* | 3600 | 41.5i | 1.7 | 3.2 |  |  | [67] | [34] | [67] | [67] |
| *Eudyptes chrysolophus* | 3400 | 41.5i | 1.5 | 2.7 |  |  | [67] | [34] | [67] | [67] |
| *Eudyptes chrysolophus* | 3300 | 41.5i | 1.8 | 2.8 |  |  | [67] | [34] | [67] | [67] |
| *Eudyptes chrysolophus* | 3200 | 41.5i | 2.0 | 3.0 |  |  | [67] | [34] | [67] | [67] |
| *Eudyptes chrysolophus* | 3100 | 41.5i | 1.1 | 3.1 |  |  | [67] | [34] | [67] | [67] |
| *Eudyptes chrysolophus* | 3000 | 41.5i | 2.1 | 2.8 |  |  | [67] | [34] | [67] | [67] |
| *Eudyptes chrysolophus* | 3000 | 41.5i | 1.3 | 2.8 |  |  | [67] | [34] | [67] | [67] |
| *Eudyptes moseleyi* | 2500 | 41.5i | 0.7 |  |  |  | [68] | [34] | [68] |  |
| *Eudyptes moseleyi* | 2300 | 41.5i | 1.0 | 2.8 | *1.0* |  | [69] | [34] | [69] | [69] |
| *Eudyptes moseleyi* | 2486.7 | 41.5i | 1.3 | 3.2 |  | *3.2* | [70] | [34] | [70] | [70] |
| *Eudyptes schlegeli* | 5000 | 41.5i | 1.7 | 7.5 | *1.7* | *7.5* | [33] | [34] | [71] | [71] |
| *Eudyptula minor* | 964 | 41.5i | 0.3 |  |  |  | [72] | [34] | [72] |  |
| *Eudyptula minor* | 1020 | 41.5i |  | 1.5 |  | *1.5* | [73] | [34] |  | [73] |
| *Eudyptula minor* | 1200 | 41.5i | 0.4 | 1.5 |  |  | [74] | [34] | [74] | [74] |
| *Eudyptula minor* | 1134.8 | 41.5i | 0.5 | 1.3 | *0.5* |  | [46] | [34] | [46] | [46] |
| *Eudyptula minor* | 1280 | 41.5i | 0.6 |  |  |  | [75] | [34] | [75] |  |
| *Fratercula arctica* | 425 | 40.1 | 0.5 | 1.9 |  | *1.9* | [36] | [34] | [36] | [36] |
| *Fratercula arctica* | 500 | 40.1 | 0.4 |  | *0.4* |  | [76] | [34] | [76] |  |
| *Fratercula cirrhata* | 900 | 39.4 | 0.8 | 1.3 | *0.8* | *1.3* | [33] | [34] | [46] | [46] |
| *Fulica americana* | 700 | 40.9 | 0.1 | 0.2 | *0.1* | *0.2* | [33] | [34] | [33] | [33] |
| *Fulica atra* | 900 | 40.9ii | 0.2 |  | *0.2* |  | [33] | [34] | [77] |  |
| *Fulmarus glacialis* | 703 | 41.5i |  | 0.1 |  | *0.1* | [78] | [34] |  | [79] |
| *Gavia adamsii* | 5200 | 39.0ii | 0.9 | 1.5 | *0.9* | *1.5* | [62] | [34] | [80] | [62] |
| *Gavia adamsii* | 5000 | 39.0ii | 1.1 |  |  |  | [33] | [34] | [81] |  |
| *Gavia arctica* | 270 | 39.0 | 0.8 | 1.0 | *0.8* |  | [33] | [34] | [82] | [82] |
| *Gavia arctica* | 2400 | 39.0 |  | 2.0 |  | *2.0* | [62] | [34] |  | [62] |
| *Gavia immer* | 3800 | 39.0ii | 0.7 | 2.1 | *0.7* | *2.1* | [33] | [34] | [46] | [46] |
| *Gavia stellata* | 1800 | 39.0ii | 0.4 |  | *0.4* |  | [33] | [34] | [77] |  |
| *Gavia stellata* | 1700 | 39.0ii |  | 1.5 |  | *1.5* | [62] | [34] |  | [62] |
| *Histrionicus histrionicus* | 800 | 41.5i | 0.3 |  | *0.3* |  | [33] | [34] | [83] |  |
| *Leucocarbo (Phal.) carunculatus* | 2500 | 41.5i | 2.1 | 3.2 | *2.1* | *3.2* | [33] | [34] | [84] | [84] |
| *Macronectes giganteus* | 3900 | 40.6 | 0.1 | 0.1 | *0.1* | *0.1* | [34] | [34] | [85] | [85] |
| *Megadyptes antipodes* | 5500 | 37.8 | 1.7 |  | *1.7* |  | [46] | [34] | [46] |  |
| *Melanitta fusca* | 1500 | 41.5i |  | 3.0 |  | *3.0* | [62] | [34] |  | [62] |
| *Melanitta nigra* | 1000 | 41.5i | 0.4 | 0.8 | *0.4* | *0.8* | [33] | [34] | [33] | [33] |
| *Melanitta perspicillata* | 1000 | 41.5i | 0.9 | 1.1 | *0.9* | *1.1* | [62] | [34] | [62] | [62] |
| *Mergus albellus* | 700 | 41.9ii | 0.3 | 0.8 | *0.3* | *0.8* | [62] | [34] | [63] | [62] |
| *Mergus merganser* | 1500 | 41.9ii | 0.4 | 2.0 | *0.4* | *2.0* | [62] | [34] | [63] | [62] |
| *Mergus serrator* | 1100 | 41.9 | 0.5 | 2.0 | *0.5* | *2.0* | [62] | [34] | [62] | [62] |
| *Morus bassanus* | 3000 | 41.5i | 0.1 | 0.7 | *0.1* | *0.7* | [46] | [34] | [46] | [46] |
| *Morus capensis* | 3000 | 41.5i | 0.1 | 0.5 | *0.1* | *0.5* | [86] | [34] | [46] | [46] |
| *Oxyura australis* | 800 | 42.2ii | 0.4 |  | *0.4* |  | [33] | [34] | [52] |  |
| *Oxyura dominica* | 400 | 42.2ii | 0.4 | 0.4 | *0.4* | *0.4* | [33] | [34] | [87] | [87] |
| *Oxyura jamaicensis* | 600 | 42.2 | 0.3 | 0.4 | *0.3* | *0.4* | [33] | [34] | [88] | [88] |
| *Oxyura leucocephala* | 700 | 42.2ii |  | 0.7 |  | *0.7* | [62] | [34] |  | [82] |
| *Pelecanoides urinatrix* | 150.5 | 41.5i | 0.3 | 0.4 | *0.3* | *0.4* | [46] | [34] | [46] | [46] |
| *Pelecanus occidentalis thagus* | 3740 | 42.2 | 0.0 |  | *0.0* |  | [34] | [34] | [89] |  |
| *Phalacororax capillatus - filamentosus)* | 2816.7 | 40.7ii | 0.7 |  |  |  | [90] | [34] | [90] |  |
| *Phalacororax capillatus - filamentosus)* | 2816 | 40.7ii | 0.5 |  |  |  | [91] | [34] | [91] |  |
| *Phalacororax capillatus - filamentosus)* | 3200 | 40.7ii | 0.5 |  | *0.5* |  | [92] | [34] | [92] |  |
| *Phalacororax capillatus - filamentosus)* | 2700 | 40.7ii | 0.4 |  |  |  | [92] | [34] | [92] |  |
| *Phalacororax capillatus - filamentosus)* | 3150 | 40.7ii | 0.6 | 1.7 |  |  | [93] | [34] | [93] | [93] |
| *Phalacororax capillatus - filamentosus)* | 3150 | 40.7ii | 0.8 | 1.8 |  |  | [93] | [34] | [93] | [93] |
| *Phalacororax capillatus - filamentosus)* | 3150 | 40.7ii | 0.6 | 1.8 |  |  | [93] | [34] | [93] | [93] |
| *Phalacororax capillatus - filamentosus)* | 3050 | 40.7ii | 0.5 | 1.5 |  |  | [93] | [34] | [93] | [93] |
| *Phalacororax capillatus - filamentosus)* | 3000 | 40.7ii | 0.6 | 2.4 |  | *2.4* | [93] | [34] | [93] | [93] |
| *Phalacororax capillatus - filamentosus)* | 2700 | 40.7ii | 0.4 | 1.0 |  |  | [93] | [34] | [93] | [93] |
| *Phalacororax capillatus - filamentosus)* | 2700 | 40.7ii | 0.4 | 0.9 |  |  | [93] | [34] | [93] | [93] |
| *Phalacororax capillatus - filamentosus)* | 2500 | 40.7ii | 0.3 | 0.8 |  |  | [93] | [34] | [93] | [93] |
| *Phalacororax capillatus - filamentosus)* | 2350 | 40.7ii | 0.4 | 1.2 |  |  | [93] | [34] | [93] | [93] |
| *Phalacororax capillatus - filamentosus)* | 2300 | 40.7ii | 0.5 | 1.3 |  |  | [93] | [34] | [93] | [93] |
| *Phalacrocorax africanus* | 577 | 40.7ii | 0.2 | 0.7 | *0.2* | *0.7* | [94] | [34] | [94] | [94] |
| *Phalacrocorax aristotelis* | 1754 | 40.7ii | 0.9 | 2.7 | *0.9* | *2.7* | [46] | [34] | [46] | [46] |
| *Phalacrocorax aristotelis* | 1741 | 40.7ii | 0.7 | 1.7 |  |  | [94] | [34] | [94] | [94] |
| *Phalacrocorax aristotelis* | 1903 | 40.7ii | 1.1 |  |  |  | [95] | [34] | [95] |  |
| *Phalacrocorax aristotelis* | 1597 | 40.7ii | 1.0 |  |  |  | [95] | [34] | [95] |  |
| *Phalacrocorax auritus* | 1700 | 41.3 | 0.4 | 0.6 | *0.4* | *0.6* | [34] | [34] | [94] | [96] |
| *Phalacrocorax bransfieldensis* | 2750 | 40.7ii | 0.9 | 2.9 | *0.9* | *2.9* | [33] | [34] | [46] | [46] |
| *Phalacrocorax brasilianus (previously olivaceus)* | 1680 | 40.7ii | 0.1 |  | *0.1* |  | [94] | [34] | [94] |  |
| *Phalacrocorax brasilianus (previously olivaceus)* | 1650 | 40.7ii | 0.3 | 0.7 | *0.3* | *0.7* | [97] | [34] | [97] | [97] |
| *Phalacrocorax capensis* | 1204 | 40.7ii | 0.4 |  | *0.4* |  | [94] | [34] | [94] |  |
| *Phalacrocorax carbo* | 3504 | 39.8 | 0.4 |  |  |  | [73] | [34] | [73] |  |
| *Phalacrocorax carbo* | 3500 | 39.8 | 0.7 | 1.0 |  |  | [98] | [34] | [98] | [98] |
| *Phalacrocorax carbo* | 3480 | 39.8 | 0.9 |  |  |  | [94] | [34] | [94] |  |
| *Phalacrocorax carbo* | 3240 | 39.8 | 0.3 |  |  |  | [75] | [34] | [75] |  |
| *Phalacrocorax carbo* | 2750 | 39.8 | 0.4 |  | *0.4* |  | [73] | [34] | [73] |  |
| *Phalacrocorax carbo* | 2630 | 39.8 | 0.3 |  |  |  | [75] | [34] | [75] |  |
| *Phalacrocorax carbo* | 2626 | 39.8 | 0.4 |  |  |  | [94] | [34] | [94] |  |
| *Phalacrocorax carbo* | 2127 | 39.8 | 0.5 | 1.2 |  |  | [94] | [34] | [94] | [94] |
| *Phalacrocorax carbo* | 2750 | 39.8 | 0.5 | 2.5 |  | *2.5* | [46] | [34] | [46] | [46] |
| *Phalacrocorax coronatus* | 760 | 40.7ii | 0.4 | 1.0 | *0.4* | *1.0* | [94] | [34] | [94] | [94] |
| *Phalacrocorax featherstoni* | 1000 | 40.7ii | 0.5 |  | *0.5* |  | [33] | [34] | [33] |  |
| *Phalacrocorax georgianus* | 2703 | 40.7ii | 3.3 | 5.2 |  |  | [99] | [34] | [99] | [99] |
| *Phalacrocorax georgianus* | 2390 | 40.7ii | 1.4 | 4.8 |  |  | [100] | [34] | [100] | [100] |
| *Phalacrocorax georgianus* | 2546 | 40.7ii | 2.7 | 6.3 | *2.7* | *6.3* | [101] | [34] | [101] | [101] |
| *Phalacrocorax lucidus* | 2884 | 40.7ii | 0.7 | 0.9 |  | *0.9* | [94] | [34] | [94] | [94] |
| *Phalacrocorax lucidus* | 2592 | 40.7ii | 0.2 | 0.5 |  |  | [94] | [34] | [94] | [94] |
| *Phalacrocorax lucidus* | 2738 | 40.7ii | 0.4 |  | *0.4* |  | [38] | [34] | [38] |  |
| *Phalacrocorax magellanicus* | 1475 | 40.7ii | 0.8 |  | *0.8* |  | [102] | [34] | [102] |  |
| *Phalacrocorax magellanicus* | 1475 | 40.7ii | 0.8 | 2.9 |  | *2.9* | [103] | [34] | [103] | [103] |
| *Phalacrocorax magellanicus* | 1475 | 40.7ii | 0.5 |  |  |  | [104] | [34] | [104] |  |
| *Phalacrocorax melanogenis* | 2454 | 40.7ii | 2.0 | 6.2 |  | *6.2* | [105] | [34] | [105] | [105] |
| *Phalacrocorax melanogenis* | 2230 | 40.7ii | 0.7 | 1.5 |  |  | [94] | [34] | [94] | [94] |
| *Phalacrocorax melanoleucos* | 780 | 40.7ii | 0.2 |  |  |  | [106] | [34] | [106] |  |
| *Phalacrocorax melanoleucos* | 731 | 40.7ii | 0.3 |  | *0.3* |  | [94] | [34] | [94] |  |
| *Phalacrocorax melanoleucos* | 1808 | 40.7ii | 0.3 |  |  |  | [107] | [34] | [107] |  |
| *Phalacrocorax neglectus* | 1945 | 40.7ii | 0.7 | 1.1 |  | *1.1* | [94] | [34] | [94] | [94] |
| *Phalacrocorax neglectus* | 1922 | 40.7ii | 0.5 | 0.6 | *0.5* |  | [94] | [34] | [94] | [94] |
| *Phalacrocorax pelagicus* | 1868 | 40.7ii | 0.7 | 1.2 | *0.7* | *1.2* | [33] | [34] | [108], [94] | [108] |
| *Phalacrocorax penicillatus* | 2500 | 40.7ii | 0.8 | 1.6 | *0.8* | *1.6* | [33] | [34] | [108], [94] | [108] |
| *Phalacrocorax punctatus* | 1275 | 40.7ii | 0.5 |  | *0.5* |  | [94] | [34] | [94] |  |
| *Phalacrocorax purpurascens* | 3200 | 40.7ii | 2.5 | 3.3 |  |  | [109] | [34] | [109] | [109] |
| *Phalacrocorax purpurascens* | 3100 | 40.7ii | 2.4 | 3.4 |  |  | [109] | [34] | [109] | [109] |
| *Phalacrocorax purpurascens* | 3000 | 40.7ii | 1.5 | 3.0 |  |  | [109] | [34] | [109] | [109] |
| *Phalacrocorax purpurascens* | 2910 | 40.7ii | 1.9 | 4.0 |  | *4.0* | [110] | [34] | [110] | [110] |
| *Phalacrocorax purpurascens* | 2900 | 40.7ii | 1.8 |  | *1.8* |  | [92] | [34] | [92] |  |
| *Phalacrocorax purpurascens* | 2520 | 40.7ii | 0.5 | 1.7 |  |  | [110] | [34] | [110] | [110] |
| *Phalacrocorax purpurascens* | 2500 | 40.7ii | 1.1 |  |  |  | [92] | [34] | [92] |  |
| *Phalacrocorax purpurascens* | 2480 | 40.7ii | 1.6 | 2.3 |  |  | [110] | [34] | [110] | [110] |
| *Phalacrocorax pygmaeus* | 700 | 40.7ii | 0.6 | 0.7 | *0.6* | *0.7* | [94] | [34] | [94] | [94] |
| *Phalacrocorax sulcirostris* | 865 | 40.7ii | 0.3 |  | *0.3* |  | [106] | [34] | [106] |  |
| *Phalacrocorax varius* | 1970 | 40.7ii | 0.4 |  |  |  | [106] | [34] | [106] |  |
| *Phalacrocorax varius* | 1814 | 40.7ii | 0.5 | 1.1 |  | *1.1* | [94] | [34] | [94] | [94] |
| *Phalacrocorax varius* | 1892 | 40.7ii | 0.4 |  | *0.4* |  | [107] | [34] | [107] |  |
| *Phalacrocorax verrucosus* | 3218 | 40.7ii |  | 6.7 |  | *6.7* | [111] | [34] |  | [111] |
| *Phalacrocorax verrucosus* | 2800 | 40.7ii |  | 5.6 |  |  | [111] | [34] |  | [111] |
| *Phalacrocorax verrucosus* | 2614 | 40.7ii |  | 5.0 |  |  | [111] | [34] |  | [111] |
| *Phalacrocorax verrucosus* | 1994 | 40.7ii |  | 3.3 |  |  | [111] | [34] |  | [111] |
| *Phalacrocorax verrucosus* | 1842 | 40.7ii |  | 3.3 |  |  | [111] | [34] |  | [111] |
| *Phalacrocorax verrucosus* | 1552 | 40.7ii |  | 1.8 |  |  | [111] | [34] |  | [111] |
| *Podiceps auritus* | 400 | 40.5 | 0.4 | 0.7 | *0.4* | *0.7* | [33] | [34] | [33] | [33] |
| *Podiceps cristatus* | 1000 | 40.4ii | 0.4 | 1.0 | *0.4* | *1.0* | [33] | [34] | [33] | [33] |
| *Podiceps grisegena* | 1100 | 40.4ii | 0.4 | 0.7 | *0.4* | *0.7* | [33] | [34] | [46] | [46] |
| *Podilymbus (Podiceps) nigricollis* | 300 | 40.5 | 0.4 | 0.8 | *0.4* | *0.8* | [62] | [34] | [33] | [62] |
| *Podilymbus (Podiceps) podiceps* | 400 | 39.3 | 0.3 | 0.5 | *0.3* | *0.5* | [33] | [34] | [33] | [33] |
| *Podilymbus (Podiceps) ruficollis* | 200 | 40.4ii | 0.2 |  | *0.2* |  | [33] | [34] | [33] |  |
| *Podilymbus gigas* | 830 | 39.3ii | 0.3 |  |  |  | [112] | [34] | [112] |  |
| *Podilymbus gigas* | 568 | 39.3ii | 0.4 |  | *0.4* |  | [112] | [34] | [112] |  |
| *Poliocephalus poliocephalus* | 250 | 42.0ii | 0.3 | 0.4 | *0.3* | *0.4* | [46] | [34] | [46] | [46] |
| *Procellaria aequinoctialis* | 1270 | 40.8 | 0.0 | 0.1 | *0.0* | *0.1* | [113] | [34] | [113] | [113] |
| *Ptychorhamphus aleuticus* | 188 | 41.5i | 0.4 | 1.2 | *0.4* | *1.2* | [46] | [34] | [46] | [46] |
| *Puffinus griseus* | 800 | 39.4ii | 0.1 |  | *0.1* |  | [33] | [34] | [46] |  |
| *Puffinus mauretanicus* | 450 | 39.4ii | 0.3 | 1.1 | *0.3* | *1.1* | [114] | [34] | [115] | [115] |
| *Puffinus tenuirostris* | 600 | 39.4ii |  | 0.2 |  | *0.2* | [33] | [34] |  | [33] |
| *Pygoscelis adeliae* | 4600 | 38.0 | 1.3 | 3.4 |  |  | [51] | [34] | [51] | [51] |
| *Pygoscelis adeliae* | 4550 | 38.0 | 1.9 | 2.3 |  |  | [116] | [34] | [116] | [116] |
| *Pygoscelis adeliae* | 4520 | 38.0 | 1.5 | 1.7 |  |  | [116] | [34] | [116] | [116] |
| *Pygoscelis adeliae* | 4266 | 38.0 | 1.2 | 1.7 |  |  | [117] | [34] | [117] | [117] |
| *Pygoscelis adeliae* | 4190 | 38.0 | 0.8 |  |  |  | [118] | [34] | [118] |  |
| *Pygoscelis adeliae* | 4040 | 38.0 | 0.5 |  |  |  | [118] | [34] | [118] |  |
| *Pygoscelis adeliae* | 4000 | 38.0 | 1.2 | 2.7 |  |  | [119] | [34] | [119] | [119] |
| *Pygoscelis adeliae* | 4000 | 38.0 | 1.4 |  |  |  | [120] | [34] | [120] |  |
| *Pygoscelis adeliae* | 4453 | 38.0 | 1.5 | 5.9 |  | *5.9* | [46] | [34] | [46] | [46] |
| *Pygoscelis antartica* | 4000 | 38.0ii | 1.5 |  | *1.5* |  | [121] | [34] | [121] |  |
| *Pygoscelis antartica* | 3800 | 38.0ii | 1.3 |  |  |  | [120] | [34] | [120] |  |
| *Pygoscelis antartica* | 3600 | 38.0ii | 1.6 | 2.6 |  |  | [122] | [34] | [122] | [122] |
| *Pygoscelis antartica* | 3100 | 38.0ii | 1.6 | 2.9 |  |  | [122] | [34] | [122] | [122] |
| *Pygoscelis antartica* | 3625 | 38.0ii | 1.2 | 3.7 |  | *3.7* | [46] | [34] | [46] | [46] |
| *Pygoscelis papua* | 5500 | 38.0ii | 1.4 |  |  |  | [120] | [34] | [120] |  |
| *Pygoscelis papua* | 5300 | 38.0ii | 2.1 | 3.2 | *2.1* |  | [121] | [34] | [121] | [121] |
| *Pygoscelis papua* | 5400 | 38.0ii | 3.0 | 6.3 |  | *6.3* | [46] | [34] | [46] | [46] |
| *Somateria mollissima* | 2100 | 41.5i | 0.6 | 1.0 | *0.6* | *1.0* | [33] | [34] | [63] | [63] |
| *Somateria spectabilis* | 1800 | 41.5i |  | 1.4 |  | *1.4* | [33] | [34] |  | [82] |
| *Spheniscus demersus* | 3000 | 41.5i | 0.8 | 2.4 |  | *2.4* | [46] | [34] | [46] | [46] |
| *Spheniscus demersus* | 3000 | 41.5i | 0.9 | 2.3 | *0.9* |  | [38] | [34] | [38] | [38] |
| *Spheniscus humboldti* | 4295 | 41.5i | 0.3 | 2.8 |  | *2.8* | [123] | [34] | [123] | [123] |
| *Spheniscus humboldti* | 4199 | 41.5i | 0.7 |  | *0.7* |  | [46] | [34] | [46] |  |
| *Spheniscus magellanicus* | 5000 | 41.5i | 0.9 |  | *0.9* |  | [33] | [34] | [46] |  |
| *Spheniscus mendiculus* | 2350 | 41.5i | 0.1 | 3.1 |  | *3.1* | [124] | [34] | [124] | [124] |
| *Spheniscus mendiculus* | 2130 | 41.5i | 0.2 | 1.9 | *0.2* |  | [124] | [34] | [124] | [124] |
| *Sula nebouxii* | 1723 | 41.0ii | 0.1 | 0.7 |  | *0.7* | [125] | [34] | [125] | [125] |
| *Sula nebouxii* | 1319 | 41.0ii | 0.1 | 0.5 | *0.1* |  | [125] | [34] | [125] | [125] |
| *Sula sula* | 915 | 41.0ii | 0.0 | 0.1 | *0.0* | *0.1* | [126] | [34] | [126] | [126] |
| *Sula variegata* | 1300 | 41.0ii | 0.1 | 0.3 | *0.1* | *0.3* | [33] | [34] | [46] | [46] |
| *Synthliboramphus hypoleucus* | 157 | 39.1 | 0.4 |  | *0.4* |  | [127] | [34] | [128] |  |
| *Tachybaptus dominicus* | 100 | 41.5i | 0.2 | 0.4 | *0.2* | *0.4* | [33] | [34] | [46] | [46] |
| *Tachybaptus novaehollandiae* | 150 | 41.5i | 0.3 | 0.4 | *0.3* | *0.4* | [129] | [34] | [129] | [129] |
| *Uria aalge* | 1092 | 41.6 | 1.1 | 3.4 | *1.1* | *3.4* | [36] | [34] | [36] | [36] |
| *Uria aalge* | 993 | 41.6 | 0.6 | 2.0 |  |  | [70] | [34] | [70] | [70] |
| *Uria aalge* | 1042 | 41.6 | 1.4 | 3.2 |  |  | [46] | [34] | [46] | [46] |
| *Uria lomvia* | 1150 | 39.0 | 0.9 | 3.7 |  |  | [130] | [34] | [130] | [130] |
| *Uria lomvia* | 1024 | 39.0 | 1.8 | 3.3 | *1.8* |  | [131] | [34] | [131] | [131] |
| *Uria lomvia* | 945 | 39.0 | 1.9 |  |  |  | [132] | [34] | [132] |  |
| *Uria lomvia* | 934 | 39.0 | 2.1 | 4.0 |  |  | [133] | [34] | [133] | [133] |
| *Uria lomvia* | 906 | 39.0 | 1.4 |  |  |  | [132] | [34] | [132] |  |
| *Uria lomvia* | 1000 | 39.0 | 1.6 | 4.1 |  | *4.1* | [46] | [34] | [46] | [46] |
|  |  |  |  |  |  |  |  |  |  |  |
| MAMMALS |  |  |  |  |  |  |  |  |  |  |
| *Aonyx capensis* | 13000 | 36.4^[[3]](#endnote-3)^ | 0.4 |  | *0.4* |  | [134] | [34] | [134] |  |
| *Arctocephalus australis* | 35000 | 36.4iii | 2.8 | 7.1 | *2.8* | *7.1* | [33] | [34] | [135] | [135] |
| *Arctocephalus forsteri Female* | 40291 | 36.4iii | 2.5 | 11.2 | *2.5* | *11.2* | [136] | [34] | [136] | [136] |
| *Arctocephalus forsteri Juveniles* | 15300 | 36.4iii | 0.4 | 1.0 |  |  | [137] | [34] | [137] | [137] |
| *Arctocephalus forsteri Juveniles* | 18000 | 36.4iii | 0.5 | 2.3 |  |  | [137] | [34] | [137] | [137] |
| *Arctocephalus forsteri Juveniles* | 16600 | 36.4iii | 0.4 | 2.9 |  |  | [137] | [34] | [137] | [137] |
| *Arctocephalus forsteri Juveniles* | 15900 | 36.4iii | 0.3 | 0.9 |  |  | [137] | [34] | [137] | [137] |
| *Arctocephalus forsteri Juveniles* | 15800 | 36.4iii | 0.5 | 3.2 |  |  | [137] | [34] | [137] | [137] |
| *Arctocephalus forsteri Juveniles* | 18500 | 36.4iii | 0.4 | 1.3 | *0.4* |  | [137] | [34] | [137] | [137] |
| *Arctocephalus forsteri Juveniles* | 22600 | 36.4iii | 0.3 | 1.3 |  |  | [137] | [34] | [137] | [137] |
| *Arctocephalus forsteri Juveniles* | 18900 | 36.4iii | 0.3 | 1.1 |  |  | [137] | [34] | [137] | [137] |
| *Arctocephalus forsteri Juveniles* | 17500 | 36.4iii | 0.4 | 1.6 |  |  | [137] | [34] | [137] | [137] |
| *Arctocephalus forsteri Juveniles* | 14500 | 36.4iii | 0.4 | 1.9 |  |  | [137] | [34] | [137] | [137] |
| *Arctocephalus forsteri Juveniles* | 18800 | 36.4iii | 0.4 | 1.5 |  |  | [137] | [34] | [137] | [137] |
| *Arctocephalus forsteri Juveniles* | 19600 | 36.4iii | 0.4 | 1.8 |  |  | [137] | [34] | [137] | [137] |
| *Arctocephalus forsteri Juveniles* | 17900 | 36.4iii | 0.4 | 1.7 |  |  | [137] | [34] | [137] | [137] |
| *Arctocephalus forsteri Juveniles* | 19600 | 36.4iii | 0.3 | 1.5 |  |  | [137] | [34] | [137] | [137] |
| *Arctocephalus forsteri Juveniles* | 16000 | 36.4iii | 0.3 | 3.3 |  | *3.3* | [137] | [34] | [137] | [137] |
| *Arctocephalus forsteri Juveniles* | 17500 | 36.4iii | 0.5 | 2.4 |  |  | [137] | [34] | [137] | [137] |
| *Arctocephalus forsteri Juveniles* | 19400 | 36.4iii | 0.3 | 1.3 |  |  | [137] | [34] | [137] | [137] |
| *Arctocephalus forsteri Juveniles* | 16500 | 36.4iii | 0.5 | 2.8 |  |  | [137] | [34] | [137] | [137] |
| *Arctocephalus forsteri Juveniles* | 17600 | 36.4iii | 0.6 | 2.3 |  |  | [137] | [34] | [137] | [137] |
| *Arctocephalus forsteri Juveniles* | 16300 | 36.4iii | 0.4 | 2.2 |  |  | [137] | [34] | [137] | [137] |
| *Arctocephalus forsteri Juveniles* | 18500 | 36.4iii | 0.4 | 2.4 |  |  | [137] | [34] | [137] | [137] |
| *Arctocephalus forsteri Male* | 106400 | 36.4iii | 6.0 | 10.8 | *6.0* | *10.8* | [138] | [34] | [138] | [138] |
| *Arctocephalus galapagoensis Females* | 28830 | 36.4iii | 2.8 | 7.7 | *2.8* | *7.7* | [139] | [34] | [140] | [140] |
| *Arctocephalus galapagoensis Juveniles* | 10030 | 36.4iii | 0.1 | 1.0 |  |  | [139] | [34] | [139] | [139] |
| *Arctocephalus galapagoensis Juveniles* | 9560 | 36.4iii | 0.7 | 3.1 | *0.7* |  | [139] | [34] | [139] | [139] |
| *Arctocephalus galapagoensis Juveniles* | 10600 | 36.4iii | 0.6 | 3.3 |  |  | [139] | [34] | [139] | [139] |
| *Arctocephalus galapagoensis Juveniles* | 14850 | 36.4iii | 1.0 | 3.3 |  |  | [139] | [34] | [139] | [139] |
| *Arctocephalus galapagoensis Juveniles* | 15040 | 36.4iii | 0.9 | 3.8 |  |  | [139] | [34] | [139] | [139] |
| *Arctocephalus galapagoensis Juveniles* | 12980 | 36.4iii | 0.6 | 5.8 |  | *5.8* | [139] | [34] | [139] | [139] |
| *Arctocephalus galapagoensis Juveniles* | 15820 | 36.4iii | 0.7 | 4.3 |  |  | [139] | [34] | [139] | [139] |
| *Arctocephalus gazella Female* | 37000 | 36.4iii | 1.0 | 10.0 |  | *10.0* | [141] | [34] | [141] | [141] |
| *Arctocephalus gazella Female* | 42500 | 36.4iii | 1.0 | 3.5 |  |  | [141] | [34] | [141] | [141] |
| *Arctocephalus gazella Female* | 46000 | 36.4iii | 1.0 | 3.7 |  |  | [141] | [34] | [141] | [141] |
| *Arctocephalus gazella Female* | 39500 | 36.4iii | 0.8 | 6.5 |  |  | [141] | [34] | [141] | [141] |
| *Arctocephalus gazella Female* | 48000 | 36.4iii | 0.8 | 3.5 |  |  | [141] | [34] | [141] | [141] |
| *Arctocephalus gazella Female* | 45000 | 36.4iii | 1.2 | 4.2 |  |  | [141] | [34] | [141] | [141] |
| *Arctocephalus gazella Female* | 42000 | 36.4iii | 1.0 | 3.5 | *1.0* |  | [141] | [34] | [141] | [141] |
| *Arctocephalus gazella Female* | 38000 | 36.4iii | 0.8 | 3.7 |  |  | [141] | [34] | [141] | [141] |
| *Arctocephalus gazella Female* | 38000 | 36.4iii | 1.0 | 3.3 |  |  | [141] | [34] | [141] | [141] |
| *Arctocephalus gazella Female* | 36500 | 36.4iii | 1.0 | 2.8 |  |  | [141] | [34] | [141] | [141] |
| *Arctocephalus gazella Female* | 40500 | 36.4iii | 0.8 | 7.3 |  |  | [141] | [34] | [141] | [141] |
| *Arctocephalus gazella Female* | 29000 | 36.4iii | 0.8 |  |  |  | [142] | [34] | [142] |  |
| *Arctocephalus gazella Female* | 34500 | 36.4iii | 0.8 |  |  |  | [142] | [34] | [142] |  |
| *Arctocephalus gazella Female* | 33000 | 36.4iii | 1.4 |  |  |  | [142] | [34] | [142] |  |
| *Arctocephalus gazella Female* | 31000 | 36.4iii | 1.2 |  |  |  | [142] | [34] | [142] |  |
| *Arctocephalus gazella Female* | 32500 | 36.4iii | 0.4 |  |  |  | [142] | [34] | [142] |  |
| *Arctocephalus gazella Female* | 39500 | 36.4iii | 1.1 |  |  |  | [142] | [34] | [142] |  |
| *Arctocephalus gazella Female* | 29500 | 36.4iii | 0.3 |  |  |  | [142] | [34] | [142] |  |
| *Arctocephalus gazella Female* | 29500 | 36.4iii | 0.8 |  |  |  | [142] | [34] | [142] |  |
| *Arctocephalus gazella Female* | 37500 | 36.4iii | 0.7 |  |  |  | [142] | [34] | [142] |  |
| *Arctocephalus gazella Female* | 35000 | 36.4iii | 0.6 |  |  |  | [142] | [34] | [142] |  |
| *Arctocephalus gazella Female* | 35900 | 36.4iii | 1.1 |  |  |  | [143] | [34] | [143] |  |
| *Arctocephalus gazella Female* | 38700 | 36.4iii | 1.1 |  |  |  | [143] | [34] | [143] |  |
| *Arctocephalus gazella Female* | 38200 | 36.4iii | 1.2 |  |  |  | [143] | [34] | [143] |  |
| *Arctocephalus gazella Female* | 43100 | 36.4iii | 1.3 |  |  |  | [144] | [34] | [144] |  |
| *Arctocephalus gazella Female* | 31725 | 36.4iii | 1.6 | 5.3 |  |  | [145] | [34] | [145] | [145] |
| *Arctocephalus gazella Female* | 37400 | 36.4iii | 0.9 | 4.7 |  |  | [146] | [34] | [146] | [146] |
| *Arctocephalus gazella Female* | 34200 | 36.4iii | 0.7 | 4.2 |  |  | [146] | [34] | [146] | [146] |
| *Arctocephalus gazella Female* | 37500 | 36.4iii | 1.7 | 4.9 |  |  | [147] | [34] | [147] | [147] |
| *Arctocephalus gazella Female* | 37500 | 36.4iii | 0.9 |  |  |  | [148] | [34] | [148] |  |
| *Arctocephalus gazella Female* | 37500 | 36.4iii | 0.6 | 5.3 |  |  | [149] | [34] | [149] | [149] |
| *Arctocephalus gazella Female* | 37500 | 36.4iii | 1.0 | 7.8 |  |  | [149] | [34] | [149] | [149] |
| *Arctocephalus gazella Male* | 90000 | 36.4iii | 1.7 | 11.0 | *1.7* | *11.0* | [150] | [34] | [150] | [150] |
| *Arctocephalus philippi Female* | 50000 | 36.4iii | 0.8 | 3.7 | *0.8* | *3.7* | [46] | [34] | [151] | [151] |
| *Arctocephalus pusillus Female* | 70500 | 36.4iii | 3.4 | 4.8 |  |  | [152] | [34] | [152] | [152] |
| *Arctocephalus pusillus Female* | 69500 | 36.4iii | 2.6 | 6.1 |  |  | [152] | [34] | [152] | [152] |
| *Arctocephalus pusillus Female* | 73500 | 36.4iii | 2.7 | 5.2 |  |  | [152] | [34] | [152] | [152] |
| *Arctocephalus pusillus Female* | 63000 | 36.4iii | 3.0 | 6.0 |  |  | [152] | [34] | [152] | [152] |
| *Arctocephalus pusillus Female* | 92000 | 36.4iii | 3.6 | 6.9 |  |  | [152] | [34] | [152] | [152] |
| *Arctocephalus pusillus Female* | 92500 | 36.4iii | 3.7 | 7.1 |  |  | [152] | [34] | [152] | [152] |
| *Arctocephalus pusillus Female* | 78500 | 36.4iii | 3.3 | 6.9 |  |  | [152] | [34] | [152] | [152] |
| *Arctocephalus pusillus Female* | 93000 | 36.4iii | 3.1 | 6.3 | *3.1* |  | [152] | [34] | [152] | [152] |
| *Arctocephalus pusillus Female* | 67000 | 36.4iii | 2.0 | 7.0 |  |  | [152] | [34] | [152] | [152] |
| *Arctocephalus pusillus Female* | 77722 | 36.4iii | 3.0 | 8.9 |  | *8.9* | [46] | [34] | [46] | [46] |
| *Arctocephalus pusillus Male* | 210000 | 36.4iii | 2.4 | 6.8 | *2.4* | *6.8* | [153] | [34] | [153] | [153] |
| *Arctocephalus townsendi Female* | 49000 | 36.4iii | 2.5 | 5.0 | *2.5* | *5.0* | [154] | [34] | [154] | [154] |
| *Arctocephalus tropicalis Females* | 31720 | 36.4iii | 1.4 | 2.4 |  |  | [155] | [34] | [155] | [155] |
| *Arctocephalus tropicalis Females* | 31700 | 36.4iii | 1.2 | 1.8 |  |  | [155] | [34] | [155] | [155] |
| *Arctocephalus tropicalis Females* | 44000 | 36.4iii | 0.9 | 4.2 |  |  | [156] | [34] | [156] | [156] |
| *Arctocephalus tropicalis Females* | 33000 | 36.4iii | 0.7 | 3.3 |  |  | [156] | [34] | [156] | [156] |
| *Arctocephalus tropicalis Females* | 55000 | 36.4iii | 1.3 | 4.8 |  |  | [156] | [34] | [156] | [156] |
| *Arctocephalus tropicalis Females* | 58000 | 36.4iii | 1.3 | 5.3 |  |  | [156] | [34] | [156] | [156] |
| *Arctocephalus tropicalis Females* | 52000 | 36.4iii | 1.1 | 3.2 |  |  | [156] | [34] | [156] | [156] |
| *Arctocephalus tropicalis Females* | 39500 | 36.4iii | 1.1 | 3.8 | *1.1* |  | [156] | [34] | [156] | [156] |
| *Arctocephalus tropicalis Females* | 46500 | 36.4iii | 0.9 | 3.8 |  |  | [156] | [34] | [156] | [156] |
| *Arctocephalus tropicalis Females* | 51000 | 36.4iii | 1.4 | 4.0 |  |  | [156] | [34] | [156] | [156] |
| *Arctocephalus tropicalis Females* | 42000 | 36.4iii | 1.0 | 2.8 |  |  | [156] | [34] | [156] | [156] |
| *Arctocephalus tropicalis Females* | 51000 | 36.4iii | 0.9 | 3.3 |  |  | [156] | [34] | [156] | [156] |
| *Arctocephalus tropicalis Females* | 47500 | 36.4iii | 0.9 | 3.5 |  |  | [156] | [34] | [156] | [156] |
| *Arctocephalus tropicalis Females* | 57000 | 36.4iii | 1.5 | 4.3 |  |  | [156] | [34] | [156] | [156] |
| *Arctocephalus tropicalis Females* | 58000 | 36.4iii | 1.2 | 4.3 |  |  | [156] | [34] | [156] | [156] |
| *Arctocephalus tropicalis Females* | 48000 | 36.4iii | 1.2 | 3.2 |  |  | [156] | [34] | [156] | [156] |
| *Arctocephalus tropicalis Females* | 56000 | 36.4iii | 0.8 | 3.3 |  |  | [156] | [34] | [156] | [156] |
| *Arctocephalus tropicalis Females* | 47000 | 36.4iii | 1.2 | 4.8 |  |  | [156] | [34] | [156] | [156] |
| *Arctocephalus tropicalis Females* | 54000 | 36.4iii | 1.8 | 4.3 |  |  | [156] | [34] | [156] | [156] |
| *Arctocephalus tropicalis Females* | 53500 | 36.4iii | 1.1 | 5.7 |  |  | [156] | [34] | [156] | [156] |
| *Arctocephalus tropicalis Females* | 59000 | 36.4iii | 2.2 | 6.5 |  | *6.5* | [156] | [34] | [156] | [156] |
| *Arctocephalus tropicalis Females* | 32700 | 36.4iii | 0.7 | 3.7 |  |  | [146] | [34] | [146] | [146] |
| *Arctocephalus tropicalis Females* | 28600 | 36.4iii | 0.7 | 3.0 |  |  | [146] | [34] | [146] | [146] |
| *Balaena mysticetus* | 79400000 | 36.4iii | 5.0 | 63.0 | *5.0* | *63.0* | [33] | [34] | [33] | [33] |
| *Balaenoptera acutorostrata* | 7500000 | 36.6^[[4]](#endnote-4)^ | 2.5 | 13.4 | *2.3* | *13.4* | [114] | [34] | [46] | [46] |
| *Balaenoptera borealis* | 23600000 | 36.6iv | 7.5 | 15.0 | *7.5* | *15.0* | [33] | [34] | [33] | [33] |
| *Balaenoptera edeni* | 20400000 | 36.6iv |  | 20.0 |  | *20.0* | [33] | [34] |  | [157] |
| *Balaenoptera musculus* | 92671000 | 36.6iv | 6.4 | 27.0 | *6.4* | *27.0* | [158] | [34] | [46] | [46] |
| *Balaenoptera physalus* | 52584000 | 36.6iv | 4.3 | 20.2 | *4.3* | *20.2* | [158] | [34] | [46] | [46] |
| *Berardius arnuxii* | 9000000 | 36.4iii | 46.4 | 153.0 | *46.4* | *153.0* | [159] | [34] | [159] | [159] |
| *Berardius bairdii* | 11000000 | 36.4iii | 17.9 | 67.0 | *17.9* | *67.0* | [46] | [34] | [46] | [46] |
| *Callorhinus ursinus females* | 36800 | 36.4iii | 2.2 | 4.8 | *2.2* |  | [160] | [34] | [160] | [160] |
| *Callorhinus ursinus females* | 45500 | 36.4iii | 2.3 | 6.0 |  |  | [160] | [34] | [160] | [160] |
| *Callorhinus ursinus females* | 41150 | 36.4iii | 1.5 | 7.6 |  | *7.6* | [46] | [34] | [46] | [46] |
| *Callorhinus ursinus Juveniles Males* | 38000 | 36.4iii | 1.5 | 5.4 |  |  | [161] | [34] | [161] | [161] |
| *Callorhinus ursinus Juveniles Males* | 51500 | 36.4iii | 1.7 | 7.3 |  |  | [161] | [34] | [161] | [161] |
| *Callorhinus ursinus Juveniles Males* | 50000 | 36.4iii | 1.2 | 8.5 |  |  | [161] | [34] | [161] | [161] |
| *Callorhinus ursinus Juveniles Males* | 71500 | 36.4iii | 1.9 | 6.8 |  |  | [161] | [34] | [161] | [161] |
| *Callorhinus ursinus Juveniles Males* | 38000 | 36.4iii | 1.3 | 9.0 |  |  | [161] | [34] | [161] | [161] |
| *Callorhinus ursinus Juveniles Males* | 44500 | 36.4iii | 1.1 | 5.8 |  |  | [161] | [34] | [161] | [161] |
| *Callorhinus ursinus Juveniles Males* | 56500 | 36.4iii | 1.2 | 7.1 |  |  | [161] | [34] | [161] | [161] |
| *Callorhinus ursinus Juveniles Males* | 29500 | 36.4iii | 1.5 | 6.7 |  |  | [161] | [34] | [161] | [161] |
| *Callorhinus ursinus Juveniles Males* | 28500 | 36.4iii | 1.6 | 9.9 |  | *9.9* | [161] | [34] | [161] | [161] |
| *Callorhinus ursinus Juveniles Males* | 59500 | 36.4iii | 1.8 | 9.3 |  |  | [161] | [34] | [161] | [161] |
| *Callorhinus ursinus Juveniles Males* | 47500 | 36.4iii | 1.3 | 7.3 | *1.3* |  | [161] | [34] | [161] | [161] |
| *Callorhinus ursinus Juveniles Males* | 27000 | 36.4iii | 1.1 | 5.3 |  |  | [161] | [34] | [161] | [161] |
| *Callorhinus ursinus Juveniles Males* | 25500 | 36.4iii | 0.6 | 3.9 |  |  | [161] | [34] | [161] | [161] |
| *Callorhinus ursinus Juveniles Males* | 51500 | 36.4iii | 1.4 | 6.5 |  |  | [161] | [34] | [161] | [161] |
| *Callorhinus ursinus Juveniles Males* | 43000 | 36.4iii | 1.4 | 3.6 |  |  | [161] | [34] | [161] | [161] |
| *Callorhinus ursinus Juveniles Males* | 37000 | 36.4iii | 0.8 | 3.9 |  |  | [161] | [34] | [161] | [161] |
| *Callorhinus ursinus Juveniles Males* | 47000 | 36.4iii | 0.8 | 5.3 |  |  | [161] | [34] | [161] | [161] |
| *Callorhinus ursinus Juveniles Males* | 48000 | 36.4iii | 0.7 | 5.0 |  |  | [161] | [34] | [161] | [161] |
| *Callorhinus ursinus Juveniles Males* | 28000 | 36.4iii | 0.8 | 7.0 |  |  | [161] | [34] | [161] | [161] |
| *Condylura cristata* | 51.5 | 37.7 | 0.2 | 0.8 | *0.2* | *0.8* | [162] | [34] | [162] | [162] |
| *Cystophora cristata* | 185500 | 36.4iii | 16.6 | 52.0 | *16.6* | *52.0* | [33] | [34] | [33] | [33] |
| *Delphinapterus leucas* | 1000000 | 36.4iii |  | 12.7 |  |  | [163] | [34] |  | [163] |
| *Delphinapterus leucas* | 450000 | 36.4iii |  | 15.8 |  |  | [164] | [34] |  | [164] |
| *Delphinapterus leucas* | 907000 | 36.4iii |  | 10.9 |  |  | [165] | [34] |  | [165] |
| *Delphinapterus leucas* | 645000 | 36.4iii |  | 13.3 |  |  | [165] | [34] |  | [165] |
| *Delphinapterus leucas* | 675000 | 36.4iii | 0.4 | 5.9 |  |  | [166] | [34] | [166] | [166] |
| *Delphinapterus leucas* | 670333 | 36.4iii | 13.1 | 22.9 | *13.1* | *22.9* | [46] | [34] | [46] | [46] |
| *Dugong dugon* | 475000 | 36.4iii | 2.3 | 12.3 | *2.3* | *12.3* | [167] | [34] | [168] | [168] |
| *Enhydra lutris* | 27700 | 38.5 | 1.3 | 4.0 | *1.3* | *4.0* | [169] | [34] | [46] | [46] |
| *Erignathus barbatus females* | 283000 | 37.2 |  | 25.0 |  | *25.0* | [170] | [34] |  | [170] |
| *Erignathus barbatus females* | 380000 | 37.2 | 1.8 | 9.2 |  |  | [171] | [34] | [171] | [171] |
| *Erignathus barbatus females* | 266000 | 37.2 | 1.5 | 9.7 |  |  | [171] | [34] | [171] | [171] |
| *Erignathus barbatus females* | 300000 | 37.2 | 2.0 | 16.5 | *2.0* |  | [171] | [34] | [171] | [171] |
| *Erignathus barbatus females* | 365000 | 37.2 | 2.7 | 18.7 |  | *18.7* | [171] | [34] | [171] | [171] |
| *Erignathus barbatus Juveniles* | 85070 | 37.2 |  | 15.0 |  | *15.0* | [33] | [34] |  | [172] |
| *Eschrichtus robustus* | 31800000 | 36.4iii | 3.0 | 11.5 | *3.0* | *11.5* | [33] | [34] | [33] | [33] |
| *Eubalaena glacialis* | 75000000 | 36.4iii | 2.4 | 22.7 | *2.4* | *22.7* | [33] | [34] | [173] | [173] |
| *Eumetopias jubatus* | 273000 | 36.4iii | 1.7 | 8.0 | *1.7* |  | [33] | [34] | [174] | [174] |
| *Globicephala macrorhynchus* | 1500000 | 35.8iv | 1.6 | 12.8 | *1.6* | *12.8* | [46] | [34] | [46] | [46] |
| *Globicephala melas* | 1500000 | 35.8iv | 4.6 | 28.0 | *4.6* | *28.0* | [46] | [34] | [46] | [46] |
| *Halichoerus grypus Females* | 159600 | 36.4iii |  | 10.0 |  |  | [175] | [34] |  | [175] |
| *Halichoerus grypus Females* | 237000 | 36.4iii | 1.8 | 9.2 |  |  | [176] | [34] | [176] | [176] |
| *Halichoerus grypus Females* | 170000 | 36.4iii | 1.9 | 4.8 | *1.9* |  | [176] | [34] | [176] | [176] |
| *Halichoerus grypus Females* | 218000 | 36.4iii | 2.4 | 7.7 |  |  | [176] | [34] | [176] | [176] |
| *Halichoerus grypus Females* | 171000 | 36.4iii | 0.9 | 3.5 |  |  | [176] | [34] | [176] | [176] |
| *Halichoerus grypus Females* | 212000 | 36.4iii | 1.7 | 4.7 |  |  | [176] | [34] | [176] | [176] |
| *Halichoerus grypus Females* | 208000 | 36.4iii | 0.3 | 0.8 |  |  | [176] | [34] | [176] | [176] |
| *Halichoerus grypus Females* | 171000 | 36.4iii | 1.6 | 4.7 |  |  | [176] | [34] | [176] | [176] |
| *Halichoerus grypus Females* | 172030 | 36.4iii | 5.5 | 22.0 |  | *22.0* | [177] | [34] | [177] | [177] |
| *Halichoerus grypus Females* | 210710 | 36.4iii | 6.7 |  |  |  | [178] | [34] | [178] |  |
| *Halichoerus grypus Females* | 210130 | 36.4iii | 6.5 |  |  |  | [179] | [34] | [179] |  |
| *Halichoerus grypus Males* | 243960 | 36.4iii | 4.9 | 20.3 | *4.9* | *20.3* | [177] | [34] | [177] | [177] |
| *Halichoerus grypus Males* | 305120 | 36.4iii | 5.4 |  |  |  | [178] | [34] | [178] |  |
| *Halichoerus grypus Males* | 306610 | 36.4iii | 4.7 |  |  |  | [179] | [34] | [179] |  |
| *Halichoerus grypus Males* | 300300 | 36.4iii | 3.8 |  |  |  | [180] | [34] | [180] |  |
| *Hydrurga leptonyx* | 197500 | 36.4iii | 2.0 | 9.4 | *2.0* | *9.4* | [181] | [34] | [181] | [181] |
| *Hyperodon ampullatus* | 6650000 | 36.4iii | 11.2 | 29.5 | *11.2* | *29.5* | [182] | [34] | [182] | [182] |
| *Inia geoffrensis* | 120000 | 36.4iii | 0.8 |  | *0.8* |  | [183] | [34] | [183] |  |
| *Kogia sima* | 250000 | 36.4iii | 12.9 | 52.2 | *12.9* | *52.2* | [184] | [34] | [184] | [184] |
| *Lagenorhynchus acutus* | 161000 | 36.4iii | 0.6 | 1.1 | *0.6* | *1.1* | [185] | [34] | [185] | [185] |
| *Leptonichotes wedelli Females* | 330000 | 36.4iii | 28.2 | 62.4 | *28.2* | *62.4* | [186] | [34] | [46] | [46] |
| *Leptonichotes wedelli Females* | 359800 | 36.4iii | 12.7 | 38.0 |  |  | [187] | [34] | [187] | [187] |
| *Leptonichotes wedelli Juveniles* | 168000 | 36.4iii |  | 33.0 |  | *33.0* | [188] | [34] |  | [188] |
| *Leptonichotes wedelli Juveniles* | 96500 | 36.4iii | 5.7 | 13.0 |  |  | [187] | [34] | [187] | [187] |
| *Leptonichotes wedelli Juveniles* | 132000 | 36.4iii | 7.3 | 16.0 | *7.3* |  | [187] | [34] | [187] | [187] |
| *Lipotes vexillifer* | 95000 | 36.4iii |  | 2.3 |  | *2.3* | [33] | [34] |  | [189] |
| *Lissodelphis borealis* | 97500 | 36.4iii |  | 6.3 |  | *6.3* | [33] | [34] |  | [33] |
| *Lobodon carcinophagus* | 201300 | 36.4iii | 5.3 | 10.8 | *5.3* | *10.8* | [33] | [34] | [190] | [190] |
| *Lutra lutra* | 5400 | 38.1 | 0.7 |  | *0.7* |  | [46] | [34] | [46] |  |
| *Megaptera novaeangliae* | 32700000 | 36.4iii | 6.5 | 21.1 | *3.0* | *21.1* | [191] | [34] | [46] | [46] |
| *Mesoplodon densirostris* | 900000 | 36.4iii | 27.4 | 57.4 | *27.4* | *57.4* | [46] | [34] | [46] | [46] |
| *Mesoplodon peruvianus* | 800000 | 36.4iii | 22.4 | 37.2 | *22.4* | *37.2* | [184] | [34] | [184] | [184] |
| *Mirounga angustirostris Females* | 307500 | 38.1 | 22.5 | 119.0 |  | *119.0* | [46] | [34] | [46] | [46] |
| *Mirounga angustirostris Females* | 247000 | 38.1 | 12.9 | 23.3 |  |  | [192] | [34] | [192] | [192] |
| *Mirounga angustirostris Females* | 253000 | 38.1 | 16.4 | 30.6 |  |  | [192] | [34] | [192] | [192] |
| *Mirounga angustirostris Females* | 307000 | 38.1 | 20.5 | 35.9 |  |  | [192] | [34] | [192] | [192] |
| *Mirounga angustirostris Females* | 325000 | 38.1 | 19.4 | 35.8 |  |  | [192] | [34] | [192] | [192] |
| *Mirounga angustirostris Females* | 300000 | 38.1 | 17.2 | 35.2 |  |  | [192] | [34] | [192] | [192] |
| *Mirounga angustirostris Females* | 348000 | 38.1 | 18.2 | 32.4 |  |  | [192] | [34] | [192] | [192] |
| *Mirounga angustirostris Females* | 401000 | 38.1 | 22.0 | 47.7 |  |  | [192] | [34] | [192] | [192] |
| *Mirounga angustirostris Females* | 399000 | 38.1 | 20.8 | 44.5 | *20.8* |  | [192] | [34] | [192] | [192] |
| *Mirounga angustirostris Females* | 242000 | 38.1 | 17.1 | 33.5 |  |  | [193] | [34] | [193] | [193] |
| *Mirounga angustirostris Females* | 291000 | 38.1 | 18.2 | 44.4 |  |  | [194] | [34] | [194] | [194] |
| *Mirounga angustirostris Females* | 292000 | 38.1 | 20.2 | 47.5 |  |  | [194] | [34] | [194] | [194] |
| *Mirounga angustirostris Females* | 304000 | 38.1 | 21.4 | 41.8 |  |  | [194] | [34] | [194] | [194] |
| *Mirounga angustirostris Females* | 316000 | 38.1 | 20.3 | 62.0 |  |  | [194] | [34] | [194] | [194] |
| *Mirounga angustirostris Females* | 384000 | 38.1 | 22.5 | 50.1 |  |  | [194] | [34] | [194] | [194] |
| *Mirounga angustirostris Females* | 369000 | 38.1 | 18.0 | 38.0 |  |  | [195] | [34] | [195] | [195] |
| *Mirounga angustirostris Females* | 279000 | 38.1 | 17.8 | 39.0 |  |  | [195] | [34] | [195] | [195] |
| *Mirounga angustirostris Females* | 307000 | 38.1 | 20.8 | 56.7 |  |  | [195] | [34] | [195] | [195] |
| *Mirounga angustirostris Females* | 390000 | 38.1 | 23.4 | 47.0 |  |  | [195] | [34] | [195] | [195] |
| *Mirounga angustirostris Females* | 375000 | 38.1 | 22.9 | 54.0 |  |  | [195] | [34] | [195] | [195] |
| *Mirounga angustirostris Females* | 340000 | 38.1 | 22.3 | 55.3 |  |  | [195] | [34] | [195] | [195] |
| *Mirounga angustirostris Females* | 348000 | 38.1 | 23.9 | 54.5 |  |  | [195] | [34] | [195] | [195] |
| *Mirounga angustirostris Females* | 394000 | 38.1 | 24.2 | 106.0 |  |  | [195] | [34] | [195] | [195] |
| *Mirounga angustirostris Females* | 256000 | 38.1 | 16.0 | 40.5 |  |  | [195] | [34] | [195] | [195] |
| *Mirounga angustirostris Females* | 308000 | 38.1 | 21.7 | 68.0 |  |  | [195] | [34] | [195] | [195] |
| *Mirounga angustirostris Females* | 330000 | 38.1 | 23.3 | 75.5 |  |  | [195] | [34] | [195] | [195] |
| *Mirounga angustirostris Females* | 301000 | 38.1 | 21.3 | 59.5 |  |  | [195] | [34] | [195] | [195] |
| *Mirounga angustirostris Females* | 254000 | 38.1 | 21.3 | 65.5 |  |  | [195] | [34] | [195] | [195] |
| *Mirounga angustirostris Females* | 263000 | 38.1 | 14.9 | 22.2 |  |  | [196] | [34] | [196] | [196] |
| *Mirounga angustirostris Juveniles* | 188650 | 38.1 | 12.4 |  |  |  | [197] | [34] | [197] |  |
| *Mirounga angustirostris Juveniles* | 174800 | 38.1 | 14.0 |  |  |  | [197] | [34] | [197] |  |
| *Mirounga angustirostris Juveniles* | 193280 | 38.1 | 11.8 |  |  |  | [197] | [34] | [197] |  |
| *Mirounga angustirostris Juveniles* | 128400 | 38.1 | 18.0 | 35.0 |  |  | [198] | [34] | [198] | [198] |
| *Mirounga angustirostris Juveniles* | 115400 | 38.1 | 15.8 | 45.5 |  |  | [198] | [34] | [198] | [198] |
| *Mirounga angustirostris Juveniles* | 134000 | 38.1 | 13.3 | 38.0 |  |  | [198] | [34] | [198] | [198] |
| *Mirounga angustirostris Juveniles* | 127000 | 38.1 | 14.1 | 47.0 |  |  | [198] | [34] | [198] | [198] |
| *Mirounga angustirostris Juveniles* | 133000 | 38.1 | 13.4 | 33.5 |  |  | [198] | [34] | [198] | [198] |
| *Mirounga angustirostris Juveniles* | 127000 | 38.1 | 14.0 | 29.0 | *14.0* |  | [198] | [34] | [198] | [198] |
| *Mirounga angustirostris Juveniles* | 110700 | 38.1 | 14.0 | 29.5 |  |  | [198] | [34] | [198] | [198] |
| *Mirounga angustirostris Juveniles* | 113000 | 38.1 | 13.5 | 86.0 |  | *86.0* | [198] | [34] | [198] | [198] |
| *Mirounga angustirostris Juveniles* | 155000 | 38.1 | 17.6 | 42.5 |  |  | [198] | [34] | [198] | [198] |
| *Mirounga angustirostris Juveniles* | 149000 | 38.1 | 16.1 | 37.0 |  |  | [198] | [34] | [198] | [198] |
| *Mirounga angustirostris Juveniles* | 162000 | 38.1 | 20.0 | 65.0 |  |  | [198] | [34] | [198] | [198] |
| *Mirounga angustirostris Juveniles* | 84200 | 38.1 | 12.1 | 26.5 |  |  | [198] | [34] | [198] | [198] |
| *Mirounga angustirostris Juveniles* | 93200 | 38.1 | 13.3 | 40.0 |  |  | [198] | [34] | [198] | [198] |
| *Mirounga angustirostris Juveniles* | 114900 | 38.1 | 15.3 | 31.5 |  |  | [198] | [34] | [198] | [198] |
| *Mirounga angustirostris Juveniles* | 128400 | 38.1 | 10.6 | 30.5 |  |  | [198] | [34] | [198] | [198] |
| *Mirounga angustirostris Juveniles* | 104600 | 38.1 | 13.2 | 32.5 |  |  | [198] | [34] | [198] | [198] |
| *Mirounga angustirostris Juveniles* | 88000 | 38.1 | 13.6 | 29.0 |  |  | [198] | [34] | [198] | [198] |
| *Mirounga angustirostris Juveniles* | 171000 | 38.1 | 17.5 | 28.0 |  |  | [198] | [34] | [198] | [198] |
| *Mirounga angustirostris Juveniles* | 174200 | 38.1 | 20.5 | 53.0 |  |  | [198] | [34] | [198] | [198] |
| *Mirounga angustirostris Juveniles* | 153000 | 38.1 | 17.9 | 53.5 |  |  | [198] | [34] | [198] | [198] |
| *Mirounga angustirostris Males* | 1175000 | 38.1 | 22.6 | 89.0 | *22.6* | *89.0* | [195] | [34] | [46] | [46] |
| *Mirounga leonina Females* | 402000 | 38.1 | 29.1 | 58.6 |  |  | [199] | [34] | [199] | [199] |
| *Mirounga leonina Females* | 362000 | 38.1 | 30.9 | 78.5 |  |  | [199] | [34] | [199] | [199] |
| *Mirounga leonina Females* | 342000 | 38.1 | 33.7 | 120.0 |  | *120.0* | [199] | [34] | [199] | [199] |
| *Mirounga leonina Females* | 302000 | 38.1 | 16.0 | 54.5 |  |  | [199] | [34] | [199] | [199] |
| *Mirounga leonina Females* | 422000 | 38.1 | 36.9 | 78.5 |  |  | [199] | [34] | [199] | [199] |
| *Mirounga leonina Females* | 333000 | 38.1 | 25.7 | 52.0 |  |  | [199] | [34] | [199] | [199] |
| *Mirounga leonina Females* | 387000 | 38.1 | 28.8 | 76.0 |  |  | [199] | [34] | [199] | [199] |
| *Mirounga leonina Females* | 278000 | 38.1 | 20.1 | 68.0 |  |  | [199] | [34] | [199] | [199] |
| *Mirounga leonina Females* | 304000 | 38.1 | 21.7 | 53.5 |  |  | [199] | [34] | [199] | [199] |
| *Mirounga leonina Females* | 282000 | 38.1 | 18.3 | 55.5 |  |  | [199] | [34] | [199] | [199] |
| *Mirounga leonina Females* | 339000 | 38.1 | 19.9 | 56.5 |  |  | [199] | [34] | [199] | [199] |
| *Mirounga leonina Females* | 400000 | 38.1 | 28.9 | 79.5 |  |  | [199] | [34] | [199] | [199] |
| *Mirounga leonina Females* | 430000 | 38.1 | 32.2 | 98.5 |  |  | [199] | [34] | [199] | [199] |
| *Mirounga leonina Females* | 392000 | 38.1 | 35.4 | 66.0 |  |  | [199] | [34] | [199] | [199] |
| *Mirounga leonina Females* | 295000 | 38.1 | 21.4 | 66.0 | *21.4* |  | [199] | [34] | [199] | [199] |
| *Mirounga leonina Females* | 422000 | 38.1 | 21.4 | 53.0 |  |  | [199] | [34] | [199] | [199] |
| *Mirounga leonina Females* | 462000 | 38.1 | 21.3 | 50.0 |  |  | [199] | [34] | [199] | [199] |
| *Mirounga leonina Females* | 366000 | 38.1 | 22.3 | 42.0 |  |  | [199] | [34] | [199] | [199] |
| *Mirounga leonina Females* | 425000 | 38.1 | 19.5 | 52.5 |  |  | [199] | [34] | [199] | [199] |
| *Mirounga leonina Females* | 390000 | 38.1 | 21.1 | 57.0 |  |  | [199] | [34] | [199] | [199] |
| *Mirounga leonina Females* | 344000 | 38.1 | 20.7 | 60.0 |  |  | [199] | [34] | [199] | [199] |
| *Mirounga leonina Females* | 367000 | 38.1 | 17.0 | 40.5 |  |  | [199] | [34] | [199] | [199] |
| *Mirounga leonina Females* | 298000 | 38.1 | 19.0 | 54.0 |  |  | [199] | [34] | [199] | [199] |
| *Mirounga leonina Females* | 402000 | 38.1 | 29.1 | 58.6 |  |  | [200] | [34] | [200] | [200] |
| *Mirounga leonina Females* | 362000 | 38.1 | 30.9 | 78.5 |  |  | [200] | [34] | [200] | [200] |
| *Mirounga leonina Females* | 342000 | 38.1 | 33.7 | 120.0 |  |  | [200] | [34] | [200] | [200] |
| *Mirounga leonina Females* | 320000 | 38.1 | 16.0 | 54.5 |  |  | [200] | [34] | [200] | [200] |
| *Mirounga leonina Females* | 422000 | 38.1 | 36.9 | 78.5 |  |  | [200] | [34] | [200] | [200] |
| *Mirounga leonina Females* | 295000 | 38.1 | 21.4 | 66.0 |  |  | [200] | [34] | [200] | [200] |
| *Mirounga leonina Females* | 422000 | 38.1 | 21.3 | 53.0 |  |  | [200] | [34] | [200] | [200] |
| *Mirounga leonina Females* | 462000 | 38.1 | 21.3 | 50.0 |  |  | [200] | [34] | [200] | [200] |
| *Mirounga leonina Females* | 366000 | 38.1 | 22.3 | 42.0 |  |  | [200] | [34] | [200] | [200] |
| *Mirounga leonina Females* | 425000 | 38.1 | 19.5 | 52.5 |  |  | [200] | [34] | [200] | [200] |
| *Mirounga leonina Females* | 239000 | 38.1 | 17.5 |  |  |  | [201] | [34] | [201] |  |
| *Mirounga leonina Females* | 303000 | 38.1 | 20.9 |  |  |  | [201] | [34] | [201] |  |
| *Mirounga leonina Females* | 452000 | 38.1 | 21.1 |  |  |  | [201] | [34] | [201] |  |
| *Mirounga leonina Females* | 456000 | 38.1 | 21.7 |  |  |  | [201] | [34] | [201] |  |
| *Mirounga leonina Females* | 334000 | 38.1 | 16.7 |  |  |  | [201] | [34] | [201] |  |
| *Mirounga leonina Females* | 309000 | 38.1 | 21.7 |  |  |  | [201] | [34] | [201] |  |
| *Mirounga leonina Females* | 323000 | 38.1 | 21.3 |  |  |  | [201] | [34] | [201] |  |
| *Mirounga leonina Females* | 367000 | 38.1 | 15.7 |  |  |  | [201] | [34] | [201] |  |
| *Mirounga leonina Females* | 295000 | 38.1 | 21.6 |  |  |  | [201] | [34] | [201] |  |
| *Mirounga leonina Females* | 362857 | 38.1 | 18.5 | 102.0 |  |  | [46] | [34] | [46] | [46] |
| *Mirounga leonina Males* | 1711000 | 38.1 | 25.2 | 63.0 |  |  | [199] | [34] | [199] | [199] |
| *Mirounga leonina Males* | 2122000 | 38.1 | 21.6 | 88.5 |  | *88.5* | [199] | [34] | [199] | [199] |
| *Mirounga leonina Males* | 1657000 | 38.1 | 27.6 | 62.5 |  |  | [199] | [34] | [199] | [199] |
| *Mirounga leonina Males* | 2143000 | 38.1 | 26.7 | 73.5 |  |  | [199] | [34] | [199] | [199] |
| *Mirounga leonina Males* | 1733000 | 38.1 | 22.3 | 67.5 | *22.3* |  | [199] | [34] | [199] | [199] |
| *Mirounga leonina Males* | 1275000 | 38.1 | 22.0 | 78.5 |  |  | [199] | [34] | [199] | [199] |
| *Mirounga leonina Males* | 2008000 | 38.1 | 31.9 | 59.5 |  |  | [199] | [34] | [199] | [199] |
| *Mirounga leonina Males* | 3600000 | 38.1 | 22.1 | 78.5 |  |  | [199] | [34] | [199] | [199] |
| *Mirounga leonina Males* | 3600000 | 38.1 | 22.1 | 78.5 |  |  | [200] | [34] | [200] | [200] |
| *Mirounga leonina Males* | 1711000 | 38.1 | 25.2 | 63.0 |  |  | [200] | [34] | [200] | [200] |
| *Mirounga leonina Males* | 2122000 | 38.1 | 21.6 | 88.5 |  |  | [200] | [34] | [200] | [200] |
| *Mirounga leonina Males* | 1657000 | 38.1 | 27.6 | 62.5 |  |  | [200] | [34] | [200] | [200] |
| *Monachus monachus* | 320000 | 36.4iii | 6.4 | 18.0 | *6.4* | *18.0* | [202] | [34] | [202] | [202] |
| *Monodon monoceros* | 1042857 | 36.4iii | 3.8 | 26.2 | *3.8* | *26.2* | [33] | [34] | [33] | [33] |
| *Neomys fodiens* | 16.0 | 37.3 |  | 0.2 |  | *0.2* | [203] | [34] |  | [203] |
| *Neophoca cinerea females* | 84500 | 36.4iii | 3.4 |  |  |  | [204] | [34] | [204] |  |
| *Neophoca cinerea females* | 72900 | 36.4iii | 3.0 |  |  |  | [204] | [34] | [204] |  |
| *Neophoca cinerea females* | 88200 | 36.4iii | 3.3 | 7.5 | *3.3* | *7.5* | [205] | [34] | [205] | [205] |
| *Neophoca cinerea females* | 84500 | 36.4iii | 3.6 |  |  |  | [204] | [34] | [204] |  |
| *Neophoca cinerea Juveniles* | 30000 | 36.4iii | 0.4 | 2.7 |  |  | [205] | [34] | [205] | [205] |
| *Neophoca cinerea Juveniles* | 44500 | 36.4iii | 3.2 | 5.8 |  |  | [205] | [34] | [205] | [205] |
| *Neophoca cinerea Juveniles* | 48300 | 36.4iii | 2.8 | 5.8 | *2.8* |  | [205] | [34] | [205] | [205] |
| *Neophoca cinerea Juveniles* | 65000 | 36.4iii | 2.8 | 9.0 |  | *9.0* | [205] | [34] | [205] | [205] |
| *Neophocaena phocaenoides* | 40000 | 36.4iii | 0.3 | 2.5 | *0.3* | *2.5* | [46] | [34] | [206] | [206] |
| *Odobenus rosmarus Males* | 1347250 | 36.4 | 5.6 | 24.0 | *5.6* | *24.0* | [207] | [34] | [46] | [46] |
| *Omatophoca rossi* | 180000 | 36.4iii | 8.5 | 9.8 |  | *9.8* | [46] | [34] | [46] | [46] |
| *Omatophoca rossi* | 160000 | 36.4iii | 6.5 |  | *6.5* |  | [208] | [34] | [208] |  |
| *Ondatra zibethicus* | 787.6 | 37.4 | 0.5 | 1.5 | *0.5* | *1.5* | [209] | [34] | [209] | [209] |
| *Orcaella brevirostris* | 155000 | 36.4iii | 1.9 | 7.2 | *1.9* | *7.2* | [33] | [34] | [210] | [210] |
| *Orcinus orca Females* | 5000000 | 36.4iii |  | 2.1 |  | *2.1* | [46] | [34] |  | [211] |
| *Orcinus orca Males* | 6000000 | 36.4iii | 2.8 | 7.7 | *2.8* | *7.7* | [46] | [34] | [46] | [46] |
| *Ornithorhynchus anatinus* | 1790 | 32.1 | 0.5 | 2.3 |  |  | [212] | [34] | [212] | [212] |
| *Ornithorhynchus anatinus* | 2020 | 32.1 | 0.6 |  | *0.6* |  | [213] | [34] | [213] |  |
| *Ornithorhynchus anatinus* | 1300 | 32.1 | 0.5 |  |  |  | [213] | [34] | [213] |  |
| *Ornithorhynchus anatinus* | 1300 | 32.1 | 0.6 |  |  |  | [213] | [34] | [213] |  |
| *Ornithorhynchus anatinus* | 1602 | 32.1 | 0.8 | 10.0 |  | *10.0* | [33] | [34] | [33] | [33] |
| *Otaria flavescens Females* | 120500 | 36.4iii | 1.9 | 7.2 |  |  | [214] | [34] | [214] | [214] |
| *Otaria flavescens Females* | 131000 | 36.4iii | 2.1 | 5.7 |  |  | [215] | [34] | [215] | [215] |
| *Otaria flavescens Females* | 165000 | 36.4iii | 2.3 | 7.3 |  |  | [215] | [34] | [215] | [215] |
| *Otaria flavescens Females* | 121000 | 36.4iii | 2.5 | 4.4 |  |  | [215] | [34] | [215] | [215] |
| *Otaria flavescens Females* | 120000 | 36.4iii | 2.8 | 6.5 |  |  | [215] | [34] | [215] | [215] |
| *Otaria flavescens Females* | 120000 | 36.4iii | 3.2 | 7.7 |  | *7.7* | [215] | [34] | [215] | [215] |
| *Otaria flavescens Females* | 100000 | 36.4iii | 2.3 | 5.2 | *2.3* |  | [215] | [34] | [215] | [215] |
| *Pagophilus groenlandica Females* | 131000 | 36.4iii | 8.1 | 15.0 | *8.1* | *15.0* | [216] | [34] | [216] | [216] |
| *Pagophilus groenlandica Females* | 123000 | 36.4iii | 3.2 | 13.0 |  |  | [217] | [34] | [217] | [217] |
| *Phoca hispida females* | 103000 | 36.4iii | 7.4 | 26.4 |  | *26.4* | [218] | [34] | [218] | [218] |
| *Phoca hispida females* | 80000 | 36.4iii | 6.2 | 22.6 |  |  | [218] | [34] | [218] | [218] |
| *Phoca hispida females* | 51000 | 36.4iii | 2.0 | 18.9 |  |  | [218] | [34] | [218] | [218] |
| *Phoca hispida females* | 76000 | 36.4iii | 8.1 | 21.6 |  |  | [218] | [34] | [218] | [218] |
| *Phoca hispida females* | 62500 | 36.4iii | 7.1 | 22.8 | *7.1* |  | [219] | [34] | [219] | [219] |
| *Phoca hispida Juveniles* | 39000 | 36.4iii | 1.4 | 5.4 |  |  | [218] | [34] | [218] | [218] |
| *Phoca hispida Juveniles* | 16000 | 36.4iii | 1.1 | 1.5 |  |  | [218] | [34] | [218] | [218] |
| *Phoca hispida Juveniles* | 37000 | 36.4iii | 2.2 | 11.5 |  |  | [218] | [34] | [218] | [218] |
| *Phoca hispida Juveniles* | 30000 | 36.4iii | 2.3 | 9.1 |  |  | [218] | [34] | [218] | [218] |
| *Phoca hispida Juveniles* | 17200 | 36.4iii | 1.2 | 12.0 | *1.2* | *12.0* | [217] | [34] | [217] | [217] |
| *Phoca hispida Juveniles* | 15800 | 36.4iii | 0.7 | 7.5 |  |  | [217] | [34] | [217] | [217] |
| *Phoca hispida Juveniles* | 17100 | 36.4iii | 0.9 | 5.8 |  |  | [217] | [34] | [217] | [217] |
| *Phoca hispida Males* | 95000 | 36.4iii | 2.0 | 22.3 |  |  | [218] | [34] | [218] | [218] |
| *Phoca hispida Males* | 80000 | 36.4iii | 2.6 | 23.2 |  | *23.2* | [218] | [34] | [218] | [218] |
| *Phoca hispida Males* | 57000 | 36.4iii | 8.2 | 16.8 |  |  | [218] | [34] | [218] | [218] |
| *Phoca hispida Males* | 66000 | 36.4iii | 5.5 | 13.4 |  |  | [218] | [34] | [218] | [218] |
| *Phoca hispida Males* | 48000 | 36.4iii | 5.6 | 15.2 | *5.6* |  | [218] | [34] | [218] | [218] |
| *Phoca hispida Males* | 70000 | 36.4iii | 9.0 | 21.4 |  |  | [218] | [34] | [218] | [218] |
| *Phoca hispida Males* | 61000 | 36.4iii | 4.3 | 17.1 |  |  | [219] | [34] | [219] | [219] |
| *Phoca hispida Males* | 70000 | 36.4iii | 5.6 | 21.0 |  |  | [219] | [34] | [219] | [219] |
| *Phoca hispida Males* | 58000 | 36.4iii | 4.0 | 12.3 |  |  | [219] | [34] | [219] | [219] |
| *Phoca hispida Males* | 57000 | 36.4iii | 6.3 | 18.6 |  |  | [219] | [34] | [219] | [219] |
| *Phoca sibirica females* | 54600 | 36.4iii | 5.7 |  | *5.7* |  | [220] | [34] | [220] |  |
| *Phoca sibirica females* | 54600 | 36.4iii | 5.6 |  |  |  | [220] | [34] | [220] |  |
| *Phoca sibirica females* | 72800 | 36.4iii | 5.4 |  |  |  | [220] | [34] | [220] |  |
| *Phoca sibirica females* | 72800 | 36.4iii | 8.2 |  |  |  | [220] | [34] | [220] |  |
| *Phoca sibirica Juveniles* | 28500 | 36.4iii | 4.0 |  | *4.0* |  | [221] | [34] | [221] |  |
| *Phoca vitulina Females* | 58100 | 36.4iii | 3.5 | 5.6 |  |  | [222] | [34] | [222] | [222] |
| *Phoca vitulina Females* | 66900 | 36.4iii | 5.1 | 7.0 |  |  | [222] | [34] | [222] | [222] |
| *Phoca vitulina Females* | 62600 | 36.4iii | 2.5 | 4.5 |  |  | [222] | [34] | [222] | [222] |
| *Phoca vitulina Females* | 88900 | 36.4iii | 7.0 | 10.3 |  |  | [222] | [34] | [222] | [222] |
| *Phoca vitulina Females* | 83500 | 36.4iii | 6.0 | 9.8 |  |  | [222] | [34] | [222] | [222] |
| *Phoca vitulina Females* | 78000 | 36.4iii | 1.1 | 2.5 |  |  | [223] | [34] | [223] | [223] |
| *Phoca vitulina Females* | 81000 | 36.4iii | 1.3 | 3.5 |  |  | [223] | [34] | [223] | [223] |
| *Phoca vitulina Females* | 87000 | 36.4iii | 1.5 | 3.0 |  |  | [223] | [34] | [223] | [223] |
| *Phoca vitulina Females* | 77000 | 36.4iii | 1.8 | 4.5 | *1.8* |  | [223] | [34] | [223] | [223] |
| *Phoca vitulina Females* | 79500 | 36.4iii | 1.0 | 2.8 |  |  | [223] | [34] | [223] | [223] |
| *Phoca vitulina Females* | 97000 | 36.4iii | 1.8 | 4.3 |  |  | [223] | [34] | [223] | [223] |
| *Phoca vitulina Females* | 97500 | 36.4iii | 1.1 | 17.2 |  | *17.2* | [223] | [34] | [223] | [223] |
| *Phoca vitulina Females* | 90500 | 36.4iii | 1.4 | 4.2 |  |  | [223] | [34] | [223] | [223] |
| *Phoca vitulina Females* | 104000 | 36.4iii | 1.5 | 15.7 |  |  | [223] | [34] | [223] | [223] |
| *Phoca vitulina Females* | 92000 | 36.4iii | 1.8 | 6.3 |  |  | [223] | [34] | [223] | [223] |
| *Phoca vitulina Females* | 105500 | 36.4iii | 1.2 | 2.7 |  |  | [223] | [34] | [223] | [223] |
| *Phoca vitulina Females* | 97000 | 36.4iii | 2.5 | 5.8 |  |  | [223] | [34] | [223] | [223] |
| *Phoca vitulina Females* | 84000 | 36.4iii | 1.9 | 5.8 |  |  | [223] | [34] | [223] | [223] |
| *Phoca vitulina Females* | 96000 | 36.4iii | 1.6 | 9.0 |  |  | [223] | [34] | [223] | [223] |
| *Phoca vitulina Females* | 76500 | 36.4iii | 2.0 | 8.8 |  |  | [223] | [34] | [223] | [223] |
| *Phoca vitulina Females* | 79500 | 36.4iii | 1.6 | 6.3 |  |  | [223] | [34] | [223] | [223] |
| *Phoca vitulina Females* | 87500 | 36.4iii | 2.1 | 6.3 |  |  | [223] | [34] | [223] | [223] |
| *Phoca vitulina Females* | 90000 | 36.4iii | 1.5 | 5.3 |  |  | [223] | [34] | [223] | [223] |
| *Phoca vitulina Females* | 93000 | 36.4iii | 1.8 | 6.7 |  |  | [223] | [34] | [223] | [223] |
| *Phoca vitulina Females* | 76000 | 36.4iii | 1.5 | 6.3 |  |  | [223] | [34] | [223] | [223] |
| *Phoca vitulina Females* | 84810 | 36.4iii | 1.6 | 5.8 |  |  | [224] | [34] | [224] | [224] |
| *Phoca vitulina Females* | 85905 | 36.4iii | 3.5 |  |  |  | [225] | [34] | [225] |  |
| *Phoca vitulina Juveniles* | 12000 | 36.4iii | 1.6 | 2.8 |  |  | [223] | [34] | [223] | [223] |
| *Phoca vitulina Juveniles* | 10400 | 36.4iii | 1.1 | 2.8 |  |  | [223] | [34] | [223] | [223] |
| *Phoca vitulina Juveniles* | 11700 | 36.4iii | 1.5 | 2.7 |  |  | [223] | [34] | [223] | [223] |
| *Phoca vitulina Juveniles* | 12100 | 36.4iii | 1.0 | 1.7 |  |  | [223] | [34] | [223] | [223] |
| *Phoca vitulina Juveniles* | 10500 | 36.4iii | 1.2 | 2.0 |  |  | [223] | [34] | [223] | [223] |
| *Phoca vitulina Juveniles* | 12200 | 36.4iii | 1.3 | 6.2 |  |  | [223] | [34] | [223] | [223] |
| *Phoca vitulina Juveniles* | 12200 | 36.4iii | 1.5 | 3.8 |  |  | [223] | [34] | [223] | [223] |
| *Phoca vitulina Juveniles* | 13500 | 36.4iii | 0.8 | 1.7 | *0.8* |  | [223] | [34] | [223] | [223] |
| *Phoca vitulina Juveniles* | 11500 | 36.4iii | 0.8 | 1.8 |  |  | [223] | [34] | [223] | [223] |
| *Phoca vitulina Juveniles* | 11400 | 36.4iii | 1.1 | 9.2 |  | *9.2* | [223] | [34] | [223] | [223] |
| *Phoca vitulina Juveniles* | 12300 | 36.4iii | 1.0 | 6.3 |  |  | [223] | [34] | [223] | [223] |
| *Phoca vitulina Juveniles* | 11400 | 36.4iii | 1.3 | 4.0 |  |  | [223] | [34] | [223] | [223] |
| *Phoca vitulina Juveniles* | 9700 | 36.4iii | 0.8 | 1.8 |  |  | [223] | [34] | [223] | [223] |
| *Phoca vitulina Juveniles* | 12000 | 36.4iii | 0.3 | 1.0 |  |  | [226] | [34] | [226] | [226] |
| *Phoca vitulina Juveniles* | 11500 | 36.4iii | 0.4 | 1.5 |  |  | [226] | [34] | [226] | [226] |
| *Phoca vitulina Juveniles* | 10600 | 36.4iii | 0.5 | 2.5 |  |  | [226] | [34] | [226] | [226] |
| *Phoca vitulina Juveniles* | 14100 | 36.4iii | 0.4 | 2.0 |  |  | [226] | [34] | [226] | [226] |
| *Phoca vitulina Juveniles* | 14500 | 36.4iii | 0.4 | 3.0 |  |  | [226] | [34] | [226] | [226] |
| *Phoca vitulina Juveniles* | 12200 | 36.4iii | 0.4 | 3.7 |  |  | [226] | [34] | [226] | [226] |
| *Phoca vitulina Juveniles* | 10500 | 36.4iii | 0.4 | 3.0 |  |  | [226] | [34] | [226] | [226] |
| *Phoca vitulina Juveniles* | 15400 | 36.4iii | 0.5 | 1.7 |  |  | [226] | [34] | [226] | [226] |
| *Phoca vitulina Males* | 119300 | 36.4iii | 7.2 | 14.8 |  |  | [222] | [34] | [222] | [222] |
| *Phoca vitulina Males* | 86600 | 36.4iii | 6.0 | 8.3 |  |  | [222] | [34] | [222] | [222] |
| *Phoca vitulina Males* | 62800 | 36.4iii | 3.5 | 5.0 |  |  | [222] | [34] | [222] | [222] |
| *Phoca vitulina Males* | 91400 | 36.4iii | 6.6 | 9.7 |  |  | [222] | [34] | [222] | [222] |
| *Phoca vitulina Males* | 77100 | 36.4iii | 3.5 | 8.8 |  |  | [222] | [34] | [222] | [222] |
| *Phoca vitulina Males* | 61200 | 36.4iii | 3.2 | 6.4 |  |  | [222] | [34] | [222] | [222] |
| *Phoca vitulina Males* | 86200 | 36.4iii | 4.8 | 8.5 | *4.8* |  | [222] | [34] | [222] | [222] |
| *Phoca vitulina Males* | 109800 | 36.4iii | 8.5 | 15.3 |  |  | [222] | [34] | [222] | [222] |
| *Phoca vitulina Males* | 73000 | 36.4iii | 6.0 | 9.3 |  |  | [222] | [34] | [222] | [222] |
| *Phoca vitulina Males* | 73700 | 36.4iii | 2.8 | 4.5 |  |  | [222] | [34] | [222] | [222] |
| *Phoca vitulina Males* | 101200 | 36.4iii | 8.1 | 12.8 |  |  | [222] | [34] | [222] | [222] |
| *Phoca vitulina Males* | 105600 | 36.4iii | 4.3 | 33.9 |  | *33.9* | [227] | [34] | [227] | [227] |
| *Phocartos hookeri females* | 91000 | 36.4iii | 4.1 | 8.9 |  |  | [228] | [34] | [228] | [228] |
| *Phocartos hookeri females* | 101000 | 36.4iii | 3.1 | 5.6 |  |  | [228] | [34] | [228] | [228] |
| *Phocartos hookeri females* | 98000 | 36.4iii | 3.5 | 7.1 |  |  | [228] | [34] | [228] | [228] |
| *Phocartos hookeri females* | 111500 | 36.4iii | 2.6 | 7.7 |  |  | [228] | [34] | [228] | [228] |
| *Phocartos hookeri females* | 126000 | 36.4iii | 4.1 | 11.3 |  |  | [228] | [34] | [228] | [228] |
| *Phocartos hookeri females* | 115000 | 36.4iii | 3.8 | 7.2 |  |  | [228] | [34] | [228] | [228] |
| *Phocartos hookeri females* | 129000 | 36.4iii | 3.2 | 6.9 |  |  | [228] | [34] | [228] | [228] |
| *Phocartos hookeri females* | 106000 | 36.4iii | 3.9 | 7.7 |  |  | [228] | [34] | [228] | [228] |
| *Phocartos hookeri females* | 107000 | 36.4iii | 4.7 | 8.5 |  |  | [228] | [34] | [228] | [228] |
| *Phocartos hookeri females* | 131500 | 36.4iii | 3.5 | 9.5 |  |  | [228] | [34] | [228] | [228] |
| *Phocartos hookeri females* | 107500 | 36.4iii | 4.0 | 7.7 |  |  | [228] | [34] | [228] | [228] |
| *Phocartos hookeri females* | 131000 | 36.4iii | 4.3 | 7.9 |  |  | [228] | [34] | [228] | [228] |
| *Phocartos hookeri females* | 102000 | 36.4iii | 4.1 | 7.5 |  |  | [228] | [34] | [228] | [228] |
| *Phocartos hookeri females* | 148500 | 36.4iii | 3.8 | 10.7 | *3.8* |  | [228] | [34] | [228] | [228] |
| *Phocartos hookeri females* | 83400 | 36.4iii | 4.0 |  |  |  | [229] | [34] | [229] |  |
| *Phocartos hookeri females* | 107000 | 36.4iii | 3.0 |  |  |  | [229] | [34] | [229] |  |
| *Phocartos hookeri females* | 121100 | 36.4iii | 3.9 |  |  |  | [229] | [34] | [229] |  |
| *Phocartos hookeri females* | 113200 | 36.4iii | 3.6 |  |  |  | [229] | [34] | [229] |  |
| *Phocartos hookeri females* | 122600 | 36.4iii | 3.3 |  |  |  | [229] | [34] | [229] |  |
| *Phocartos hookeri females* | 115000 | 36.4iii | 4.5 |  |  |  | [229] | [34] | [229] |  |
| *Phocartos hookeri females* | 107200 | 36.4iii | 2.6 |  |  |  | [229] | [34] | [229] |  |
| *Phocartos hookeri females* | 120600 | 36.4iii | 3.6 |  |  |  | [229] | [34] | [229] |  |
| *Phocartos hookeri females* | 105800 | 36.4iii | 4.1 |  |  |  | [229] | [34] | [229] |  |
| *Phocartos hookeri females* | 91600 | 36.4iii | 3.5 |  |  |  | [229] | [34] | [229] |  |
| *Phocartos hookeri females* | 135200 | 36.4iii | 2.5 |  |  |  | [229] | [34] | [229] |  |
| *Phocartos hookeri females* | 123000 | 36.4iii | 2.4 |  |  |  | [229] | [34] | [229] |  |
| *Phocartos hookeri females* | 106000 | 36.4iii | 3.0 |  |  |  | [230] | [34] | [230] |  |
| *Phocartos hookeri females* | 107200 | 36.4iii | 2.6 |  |  |  | [230] | [34] | [230] |  |
| *Phocartos hookeri females* | 120600 | 36.4iii | 3.3 |  |  |  | [230] | [34] | [230] |  |
| *Phocartos hookeri females* | 103600 | 36.4iii | 2.6 |  |  |  | [230] | [34] | [230] |  |
| *Phocartos hookeri females* | 105800 | 36.4iii | 4.1 |  |  |  | [230] | [34] | [230] |  |
| *Phocartos hookeri females* | 116600 | 36.4iii | 3.6 |  |  |  | [230] | [34] | [230] |  |
| *Phocartos hookeri females* | 147400 | 36.4iii | 3.4 | 11.5 |  |  | [230] | [34] | [230] | [230] |
| *Phocartos hookeri females* | 91600 | 36.4iii | 3.8 |  |  |  | [230] | [34] | [230] |  |
| *Phocartos hookeri females* | 119000 | 36.4iii | 4.4 | 8.4 |  |  | [231] | [34] | [231] | [231] |
| *Phocartos hookeri females* | 124500 | 36.4iii | 5.0 | 11.3 |  |  | [231] | [34] | [231] | [231] |
| *Phocartos hookeri females* | 109500 | 36.4iii | 3.6 | 10.8 |  |  | [231] | [34] | [231] | [231] |
| *Phocartos hookeri females* | 123500 | 36.4iii | 4.3 | 7.0 |  |  | [231] | [34] | [231] | [231] |
| *Phocartos hookeri females* | 105000 | 36.4iii | 4.5 | 10.6 |  |  | [231] | [34] | [231] | [231] |
| *Phocartos hookeri females* | 119000 | 36.4iii | 3.9 | 9.8 |  |  | [231] | [34] | [231] | [231] |
| *Phocartos hookeri females* | 113000 | 36.4iii | 4.0 | 8.8 |  |  | [231] | [34] | [231] | [231] |
| *Phocartos hookeri females* | 91500 | 36.4iii | 4.4 | 13.5 |  |  | [231] | [34] | [231] | [231] |
| *Phocartos hookeri females* | 120500 | 36.4iii | 3.6 | 9.7 |  |  | [231] | [34] | [231] | [231] |
| *Phocartos hookeri females* | 114500 | 36.4iii | 4.2 | 9.2 |  |  | [231] | [34] | [231] | [231] |
| *Phocartos hookeri females* | 103000 | 36.4iii | 3.5 | 14.5 |  | *14.5* | [231] | [34] | [231] | [231] |
| *Phocartos hookeri females* | 141500 | 36.4iii | 4.1 | 13.2 |  |  | [231] | [34] | [231] | [231] |
| *Phocartos hookeri females* | 104500 | 36.4iii | 3.6 | 9.6 |  |  | [231] | [34] | [231] | [231] |
| *Phocartos hookeri females* | 109000 | 36.4iii | 3.5 | 10.1 |  |  | [231] | [34] | [231] | [231] |
| *Phocartos hookeri females* | 133000 | 36.4iii | 4.1 | 12.9 |  |  | [231] | [34] | [231] | [231] |
| *Phocartos hookeri females* | 99000 | 36.4iii | 3.9 | 7.7 |  |  | [231] | [34] | [231] | [231] |
| *Phocartos hookeri females* | 116000 | 36.4iii | 3.4 | 8.1 |  |  | [231] | [34] | [231] | [231] |
| *Phocartos hookeri females* | 103000 | 36.4iii | 4.0 | 9.7 |  |  | [231] | [34] | [231] | [231] |
| *Phocoena phocoena Females* | 43700 | 36.4iii | 1.1 | 4.3 | *1.1* |  | [232] | [34] | [232] | [232] |
| *Phocoena phocoena Females* | 59200 | 36.4iii | 1.6 | 4.7 |  |  | [232] | [34] | [232] | [232] |
| *Phocoena phocoena Females* | 37000 | 36.4iii | 0.4 | 3.2 |  |  | [233] | [34] | [233] | [233] |
| *Phocoena phocoena Females* | 37000 | 36.4iii | 0.4 |  |  |  | [234] | [34] | [234] |  |
| *Phocoena phocoena Females* | 48000 | 36.4iii | 0.4 |  |  |  | [234] | [34] | [234] |  |
| *Phocoena phocoena Females* | 37100 | 36.4iii | 1.3 | 3.3 |  |  | [235] | [34] | [235] | [235] |
| *Phocoena phocoena Females* | 48500 | 36.4iii | 1.1 | 4.4 |  |  | [235] | [34] | [235] | [235] |
| *Phocoena phocoena Females* | 70000 | 36.4iii | 1.2 | 5.4 |  | *5.4* | [235] | [34] | [235] | [235] |
| *Phocoena phocoena Females* | 42500 | 36.4iii | 1.7 | 3.7 |  |  | [235] | [34] | [235] | [235] |
| *Phocoena phocoena Females* | 47000 | 36.4iii | 1.4 | 4.1 |  |  | [236] | [34] | [236] | [236] |
| *Phocoena phocoena Males* | 29600 | 36.4iii | 0.7 | 3.4 |  |  | [235] | [34] | [235] | [235] |
| *Phocoena phocoena Males* | 45800 | 36.4iii | 0.9 | 4.3 |  | *4.3* | [235] | [34] | [235] | [235] |
| *Phocoena phocoena Males* | 38900 | 36.4iii | 0.8 | 3.9 | *0.8* |  | [235] | [34] | [235] | [235] |
| *Phocoenoides dalli* | 50000 | 36.4iii | 1.3 | 2.8 | *1.3* | *2.8* | [237] | [34] | [237] | [237] |
| *Physeter macrocephalus Females* | 25000000 | 36.4iii | 36.2 | 40.2 | *36.2* | *40.2* | [46] | [34] | [238] | [238] |
| *Physeter macrocephalus Males* | 50000000 | 36.4iii | 21.9 | 73.0 | *21.9* | *73.0* | [46] | [34] | [239] | [239] |
| *Platanista gangetica* | 77500 | 36.4iii | 0.8 | 3.0 | *0.8* | *3.0* | [33] | [34] | [33] | [33] |
| *Sotalia fluviatilis* | 41000 | 36.4iii | 0.7 | 1.5 | *0.7* | *1.5* | [33] | [34] | [240] | [240] |
| *Stenella attenuata* | 80000 | 36.4iii | 1.7 | 5.0 |  | *5.0* | [33] | [34] | [46] | [46] |
| *Stenella frontalis* | 127000 | 36.4iii |  | 6.0 |  | *6.0* | [241] | [34] |  | [241] |
| *Stenella longirostris* | 77000 | 36.4iii | 2.3 |  | *2.3* |  | [33] | [34] | [33] |  |
| *Tursiops truncatus* | 201000 | 36.4iii | 0.7 | 4.3 | *0.7* | *4.3* | [46] | [34] | [46] | [46] |
| *Ursus maritimus* | 450000 | 36.8 | 0.2 | 0.5 | *0.2* | *0.5* | [46] | [34] | [242] | [242] |
| *Zalophus californianus* | 110100 | 36.4iii | 2.1 | 9.9 | *2.1* | *9.9* | [243] | [34] | [243] | [243] |
| *Zalophus wollebaeki* | 91000 | 36.4iii |  | 6.0 |  | *6.0* | [33] | [34] |  | [140] |
| *Ziphius cavirostris* | 2112500 | 36.4iii | 41.2 | 88.1 | *41.2* | *88.1* | [33] | [34] | [46] | [46] |


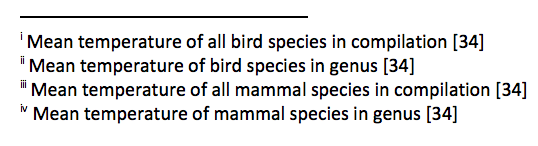


References for Appendix 1

1. Samajová P, and Gvozdík L. 2009. The influence of temperature on diving behaviour in the alpine newt, Triturus alpestris. *Journal of Thermal Biology* 34:401-405.

2. Campbell HA, Sullivan S, Read MA, Gordos MA, and Franklin CE. 2010. Ecological and physiological determinants of dive duration in the freshwater crocodile. *Functional Ecology* 24:103-111.

3. Seebacher F, Franklin CraigÂ E, and Read M. 2005. Diving Behaviour of a Reptile (Crocodylus johnstoni) in the Wild: Interactions with Heart Rate and Body Temperature. *Physiological and Biochemical Zoology* 78:1-8.

4. Grigg GC, Farwell W, Kinney J, Harlow P, Taplin L, Johansen K, and Johansen K. 1985. Diving and Amphibious Behaviour In Free-Living Crocodylus porosus. *The Australian Zoologist* 21:599-605.

5. Wright JC, Grigg GC, and Franklin CE. 1992. Redistribution of air within the lungs may potentiate "fright" bradycardia in submerged crocodiles (Crocodylus porosus). *Comparative Biochemistry and Physiology Part A: Physiology* 102:33-36.

6. Hobson ES. 1969. Remarks on Aquatic Habits of the Galapagos Marine Iguana, Including Submergence Times, Cleaning Symbiosis, and the Shark Threat. *Copeia* 1969:401-402

7. Bartholomew GA, and Lasiewski RC. 1965. Heating and cooling rates, heart rate and simulated diving in the Galapagos marine iguana. *Comparative Biochemistry and Physiology* 16:573-582.

8. Hare KM, and Miller KA. 2009. What Dives Beneath: Diving as a Measure of Performance in Lizards. *Herpetologica* 65:227-236. doi:10.1655/08-032R2.1

9. Heatwole H, and Seymour R. 1975. Pulmonary and cutaneous oxygen uptake in sea snakes and a file snake. *Comparative Biochemistry and Physiology Part A: Physiology* 51:399-405.

10. Pratt KL, Campbell HA, Watts ME, and Franklin CE. 2010. Environmental and ecological factors influencing dive behaviour in the freshwater snake Acrochordus arafurae: a field-based telemetric study. *Marine and Freshwater Research* 61:560-567.

11. Heatwole H. 1975. Voluntary submergence times of marine snakes. *Marine Biology* 32:205-213.

12. Brischoux F, Bonnet X, and Shine R. 2007. Foraging ecology of sea kraits Laticauda spp. in the Neo-Caledonian Lagoon. *Marine Ecology Progress Series* 350:145-151. 10.3354/meps07133

13. Rubinoff I, Graham J, and Motta J. 1986. Diving of the sea snake Pelamis platurus in the Gulf of Panamá. *Marine Biology* 91:181-191

14. Prassack SL, Bagatto B, and Henry RP. 2001. Effects of temperature and aquatic PO2 on the physiology and behaviour of Apalone ferox and Chrysemys picta. *J Exp Biol* 204:2185-2195.

15. Hatase H, Omuta K, and Tsukamoto K. 2007. Bottom or midwater: alternative foraging behaviours in adult female loggerhead sea turtles. *Journal of Zoology* 273:46-55

16. Hochscheid S, Bentivegna F, and Hays GC. 2005. First records of dive durations for a hibernating sea turtle. *Biology Letters* 1:82-86. 10.1098/rsbl.2004.0250

17. Hochscheid S, McMahon CR, Bradshaw CJA, Maffucci F, Bentivegna F, and Hays GC. 2007. Allometric scaling of lung volume and its consequences for marine turtle diving performance. *Comparative Biochemistry and Physiology - Part A: Molecular & Integrative Physiology* 148:360-367.

18. Houghton JDR, Broderick AC, Godley BJ, Metcalfe JD, and Hays GC. 2002. Diving behaviour during the internesting interval for loggerhead turtles Caretta caretta nesting in Cyprus. *Marine Ecology Progress Series* 227:63-70. 10.3354/meps227063

19. Minamikawa S, Naito Y, Sato K, Matsuzawa Y, Bando T, and Sakamoto W. 2000. Maintenance of neutral buoyancy by depth selection in the loggerhead turtle Caretta caretta. *J Exp Biol* 203:2967-2975.

20. Hatase H, Sato K, Yamaguchi M, Takahashi K, and Tsukamoto K. 2006. Individual variation in feeding habitat use by adult female green sea turtles Chelonia mydas: are they obligately neritic herbivores? *Oecologia* 149:52-64. 10.1007/s00442-006-0431-2

21. Hays GC, Adams CR, Broderick AC, Godley BJ, Lucas DJ, Metcalfe JD, and Prior AA. 2000. The diving behaviour of green turtles at Ascension Island. *Animal Behaviour* 59:577-586.

22. Rice MR, and Balazs GH. 2008. Diving behavior of the Hawaiian green turtle (Chelonia mydas) during oceanic migrations. *Journal of Experimental Marine Biology and Ecology* 356:121-127.

23. Southwood AL, Reina RD, Jones VS, and Jones DR. 2003. Seasonal diving patterns and body temperatures of juvenile green turtles at Heron Island, Australia. *Canadian Journal of Zoology* 81:1014-1024.

24. Southwood A, Reina R, Jones V, Speakman J, and Jones D. 2006. Seasonal metabolism of juvenile green turtles (Chelonia mydas) at Heron Island, Australia. *Canadian Journal of Zoology* 84:125-135.

25. Santos EA, Laitano SYT, and Genofre GC. 1990. Diving physiology of Chrysemys dorbignyi Dum & Bibr., 1835 (Reptilia: Chelonia). *Comparative Biochemistry and Physiology Part A: Physiology* 95:229-236.

26. Gordos M, and Franklin CE. 2002. Diving behaviour of two Australian bimodally respiring turtles, Rheodytes leukops and Emydura macquarii, in a natural setting. *Journal of Zoology* 258:335-342. 10.1017/s0952836902001474

27. Priest TE, and Franklin CE. 2002. Effect of Water Temperature and Oxygen Levels on the Diving Behavior of Two Freshwater Turtles: Rheodytes leukops and Emydura macquarii. *Journal of Herpetology* 36:555-561.

28. Blumenthal J, Austin T, Bothwell J, Broderick A, Ebanks-Petrie G, Olynik J, Orr M, Solomon J, Witt M, and Godley B. 2009. Diving behavior and movements of juvenile hawksbill turtles Eretmochelys imbricata on a Caribbean coral reef. *Coral Reefs* 28:55-65. 10.1007/s00338-008-0416-1

29. Dam RP, and Diez CF. 1996. Diving behavior of immature hawksbills (Eretmochelys imbricata) in a Caribbean cliff-wall habitat. *Marine Biology* 127:171-178. 10.1007/bf00993657

30. Gitschlag GR. 1996. Migration and diving behavior of Kemp's ridley (Garman) sea turtles along the U.S. southeastern Atlantic coast. *Journal of Experimental Marine Biology and Ecology* 205:115-135.

31. Beavers SC, and Cassano ER. 1996. Movements and Dive Behavior of a Male Sea Turtle (Lepidochelys olivacea) in the Eastern Tropical Pacific. *Journal of Herpetology* 30:97-104.

32. McMahon CR, Bradshaw CJA, and Hays GC. 2007. Satellite tracking reveals unusual diving characteristics for a marine reptile, the olive ridley turtle Lepidochelys olivacea. *Marine Ecology Progress Series* 329:239-252. 10.3354/meps329239

33. Halsey LG, Blackburn TM, and Butler PJ. 2006. A comparative analysis of the diving behaviour of birds and mammals. *Functional Ecology* 20:889-899. 10.1111/j.1365-2435.2006.01170.x

34. Clarke A, and Rothery P. 2008. Scaling of body temperature in mammals and birds. *Functional Ecology* 22:58-67.

35. Benvenuti S, Dall'Antonia L, and Lyngs P. 2001. Foraging behaviour and time allocation of chick-rearing Razorbills Alca torda at Græsholmen, central Baltic Sea. *Ibis* 143:402-412. 10.1111/j.1474-919X.2001.tb04941.x

36. Wanless S, Morris J, and Harris M. 1988. Diving behaviour of guillemot Uria aalge, puffin Fratercula arctica and razorbill Alca torda as shown by radio‐telemetry. *Journal of Zoology* 216:73-81.

37. Harding AMA, Egevang C, Walkusz W, Merkel F, Blanc S, and Grémillet D. 2009. Estimating prey capture rates of a planktivorous seabird, the little auk (Alle alle), using diet, diving behaviour, and energy consumption. *Polar Biology* 32:785-796.

38. Ryan P, Petersen S, Simeone A, and Grémillet D. 2007. Diving behaviour of African penguins: do they differ from other Spheniscus penguins? *African Journal of Marine Science* 29:153-160.

39. Van Dam R, Ponganis P, Ponganis K, Levenson D, and Marshall G. 2002. Stroke frequencies of emperor penguins diving under sea ice. *Journal of Experimental Biology* 205:3769-3774.

40. Kooyman G, and Kooyman T. 1995. Diving behavior of emperor penguins nurturing chicks at Coulman Island, Antarctica. *Condor*: 536-549.

41. Ponganis P, Stockard T, Meir J, Williams C, Ponganis K, and Howard R. 2009. O2 store management in diving emperor penguins. *Journal of Experimental Biology* 212:217-224.

42. Kirkwood R, and Robertson G. 1997. The foraging ecology of female emperor penguins in winter. *Ecological Monographs* 67:155-176.

43. Wienecke B, Robertson G, Kirkwood R, and Lawton K. 2007. Extreme dives by free-ranging emperor penguins. *Polar Biology* 30:133-142.

44. Kooyman G, Drabek C, Elsner R, and Campbell W. 1971. Diving behavior of the emperor penguin, Aptenodytes forsteri. *The Auk*:775-795.

45. Ponganis P, Van Dam R, Levenson D, Knower T, Ponganis K, and Marshall G. 2003. Regional heterothermy and conservation of core temperature in emperor penguins diving under sea ice. *Comparative Biochemistry and Physiology Part A: Molecular & Integrative Physiology* 135:477-487.

46. Ropert-Coudert Y, Kato A. (2012) The Penguiness book. World Wide Web electronic publication (http://penguinessbook.scarmarbin.be/), version 2.0, March 2012.

47. Kooyman G, Cherel Y, Maho YL, Croxall J, Thorson P, Ridoux V, and Kooyman C. 1992. Diving behavior and energetics during foraging cycles in king penguins. *Ecological Monographs* 62:143-163.

48. Moore GJ, Wienecke B, and Robertson G. 1999. Seasonal change in foraging areas and dive depths of breeding king penguins at Heard Island. *Polar Biology* 21:376-384.

49. Culik B, Pütz K, Wilson R, Allers D, Lage J, Bost C, and Le Maho Y. 1996. Diving energetics in king penguins (Aptenodytes patagonicus). *Journal of Experimental Biology* 199:973-983.

50. Pütz K, Wilson R, Charrassin J-B, Raclot T, Lage J, Le Maho Y, Kierspel M, Culik B, and Adelung D. 1998. Foraging strategy of king penguins (Aptenodytes patagonicus) during summer at the Crozet Islands. *Ecology* 79:1905-1921.

51. Sato K, Naito Y, Kato A, Niizuma Y, Watanuki Y, Charrassin J, Bost C-A, Handrich Y, and Le Maho Y. 2002. Buoyancy and maximal diving depth in penguins. *Journal of Experimental Biology* 205:1189-1197.

52. Lalas C. 1983. Comparative feeding ecology of New Zealand marine shags (Phalacrocoracidae). Ph.D. Dissertation, University of Otago, Otago.

53. Butler P, and Woakes A. 1979. Changes in heart rate and respiratory frequency during natural behaviour of ducks, with particular reference to diving. *Journal of Experimental Biology* 79:283-300.

54. Butler P, and Woakes A. 1982. Control of heart rate by carotid body chemoreceptors during diving in tufted ducks. *Journal of Applied Physiology* 53:1405-1410.

55. Lovvorn JR, and Jones DR. 1991. Body mass, volume, and buoyancy of some aquatic birds, and their relation to locomotor strategies. *Canadian Journal of Zoology* 69:2888-2892.

56. Henkel LA, Burkett EE, and Takekawa JY. 2004. At-sea activity and diving behavior of a radio-tagged Marbled Murrelet in central California. *Waterbirds* 27:9-12.

57. Jodice PG, and Collopy MW. 1999. Diving and foraging patterns of Marbled Murrelets (Brachyramphus marmoratus): testing predictions from optimal-breathing models. *Canadian Journal of Zoology* 77:1409-1418.

58. Heintzelman DS. 1963. Diving times of a common goldeneye. *The Wilson Bulletin* 75:91-91.

59. Magnusdottir M, and Einarsson A. 1990. Diving times of ducks at Lake Myvatn. *Nattutuverndarrao* 23:79-92.

60. Kuroki M, Kato A, Watanuki Y, Niizuma Y, Takahashi A, and Naito Y. 2003. Diving behavior of an epipelagically feeding alcid, the Rhinoceros Auklet (Cerorhinca monocerata). *Canadian Journal of Zoology* 81:1249-1256.

61. Burger AE, Wilson RP, Garnier D, and Wilson M-PT. 1993. Diving depths, diet, and underwater foraging of Rhinoceros Auklets in British Columbia. *Canadian Journal of Zoology* 71:2528-2540.

62. Schreer, J. F., & Kovacs, K. M. (1997). Allometry of diving capacity in air-breathing vertebrates. *Canadian Journal of Zoology*, *75*(3), 339-358.

63. Nilsson L. 1972. Habitat selection, food choice, and feeding habits of diving ducks in coastal waters of south Sweden during the non-breeding season. *Ornis Scandinavica*:55-78.

64. Hedd A, Gales R, Brothers N, and Robertson G. 1997. Diving behaviour of the Shy Albatross Diomedea cauta in Tasmania: initial findings and dive recorder assessment. *Ibis* 139:452-460.

65. Sakamoto KQ, Takahashi A, Iwata T, and Trathan PN. 2009. From the eye of the albatrosses: a bird-borne camera shows an association between albatrosses and a killer whale in the Southern Ocean. *PLoS One* 4:e7322.

66. Green J, Butler P, Woakes A, and Boyd I. 2003. Energetics of diving in macaroni penguins. *Journal of Experimental Biology* 206:43-57.

67. Sato K, Charrassin J-Bt, Bost C-A, and Naito Y. 2004. Why do macaroni penguins choose shallow body angles that result in longer descent and ascent durations? *Journal of Experimental Biology* 207:4057-4065.

68. Tremblay Y, and Cherel Y. 1999. Synchronous underwater foraging behavior in penguins. *Condor*:179-185.

69. Cherel Y, Tremblay Y, Guinard E, and Georges J. 1999. Diving behaviour of female northern rockhopper penguins, Eudyptes chrysocome moseleyi, during the brooding period at Amsterdam Island (Southern Indian Ocean). *Marine Biology* 134:375-385.

70. Tremblay Y, and Cherel Y. 2003. Geographic variation in the foraging behaviour, diet and chick growth of rockhopper penguins. *Marine Ecology Progress Series* 251:279-297.

71. Hull CL. 2000. Comparative diving behaviour and segregation of the marine habitat by breeding Royal Penguins, Eudyptes schlegeli, and eastern Rockhopper Penguins, Eudyptes chrysocome filholi, at Macquarie Island. *Canadian Journal of Zoology* 78:333-345.

72. Gales R, Williams C, and Ritz D. 1990. Foraging behaviour of the little penguin, Eudyptula minor: initial results and assessment of instrument effect. *Journal of Zoology* 220:61-85.

73. Ropert-Coudert Y, Chiaradia A, and Kato A. 2006. An exceptionally deep dive by a little penguin, Eudyptula minor. *Mar Ornithol* 34:71-74.

74. Bethge P, Nicol S, Culik B, and Wilson R. 1997. Diving behaviour and energetics in breeding little penguins (Eudyptula minor). *Journal of Zoology* 242:483-502.

75. Kato A, Ropert-Coudert Y, Grémillet D, and Cannell B. 2006. Locomotion and foraging strategy in foot-propelled and wing-propelled shallow-diving seabirds. *Marine Ecology Progress Series* 308:293-301.

76. Axelsen BE, Anker-Nilssen T, Fossum P, Kvamme C, and Nøttestad L. 2001. Pretty patterns but a simple strategy: predator-prey interactions between juvenile herring and Atlantic puffins observed with multibeam sonar. *Canadian Journal of Zoology* 79:1586-1596.

77. Dewar JM. 1924. *The bird as a diver*. Witherby, London.

78. Mallory ML, and Forbes MR. 2005. Sex discrimination and measurement bias in Northern Fulmars Fulmarus glacialis from the Canadian Arctic. *Ardea* 93:25-36.

79. Garthe S, and Furness RW. 2001. Frequent shallow diving by a Northern Fulmar feeding at Shetland. *Waterbirds*:287-289.

80. Sjölander S, and Ågren G. 1976. Reproductive behavior of the Yellow-billed Loon, Gavia adamsii. *The Condor* 78:454-463.

81. Johnsgard PA. 1987. *Diving birds of North America*: University of Nebraska Press Lincoln.

82. Cramp SS, K. E. L. 1977. *Handbook of the birds of Europe, the Middle East and North Africa*: Oxford University Press.

83. Rodway MS. 1998. Activity patterns, diet, and feeding efficiency of Harlequin Ducks breeding in northern Labrador. *Canadian Journal of Zoology* 76:902-909.

84. Brown D. 2001. Dive duration and some diving rhythms of the New Zealand king shag (Leucocarbo carunculatus). *Notornis* 48:171-177.

85. Van Den Hoff J, and Newbery K. 2006. Southern giant petrels Macronectes giganteus diving on submerged carrion. *Marine Ornithology* 34:61-64.

86. Ropert‐Coudert Y, Daunt F, Kato A, Ryan PG, Lewis S, Kobayashi K, Mori Y, Grémillet D, and Wanless S. 2009. Underwater wingbeats extend depth and duration of plunge dives in northern gannets Morus bassanus. *Journal of avian biology* 40:380-387.

87. Jenni DA. 1969. Diving times of the least grebe and masked duck. *The Auk* 86:355-356.

88. Heintzelman DS, and Newberry CJ. 1964. Some waterfowl diving times. *The Wilson Bulletin*:291-291.

89. Duffy DC. 1983. The foraging ecology of Peruvian seabirds. *The Auk*:800-810.

90. Ishikawa K, and Watanuki Y. 2002. Sex and individual differences in foraging behavior of Japanese cormorants in years of different prey availability. *Journal of Ethology* 20:49-54.

91. Kato A WY, Naito Y. 1998. Benthic and pelagic foraging of two japanese cormorants, determined by simultaneous recording of location and diving activity. *Journal of Yamashina Institute of Ornithology* 30:101-108.

92. Kato A, Watanuki Y, Shaughnessy P, Le Maho Y, and Naito Y. 1999. Intersexual differences in the diving behaviour of foraging subantarctic cormorant (Phalacrocorax albiventer) and Japanese cormorant (P. filamentosus). *Comptes Rendus de l'Académie des Sciences-Series III-Sciences de la Vie* 322:557-562.

93. Watanuki Y, Kato A, and Naito Y. 1996. Diving performance of male and female Japanese cormorants. *Canadian Journal of Zoology* 74:1098-1109.

94. Cooper J. 1986. Diving patterns of cormorants Phalacrocoracidae. *Ibis* 128:562-570.

95. Wanless S, Corfield T, Harris M, Buckland S, and Morris J. 1993. Diving behaviour of the shag Phalacrocorax aristotelis (Aves: Pelecaniformes) in relation to water depth and prey size. *Journal of Zoology* 231:11-25.

96. Ainley D, Strong C, Penniman T, and Boekelheide R. 1990. The feeding ecology of Farallon seabirds. *Seabirds of the Farallon Islands: ecology, dynamics, and structure of an upwelling-system community Stanford University Press, Stanford, California*:51-127.

97. Quintana F, Yorio P, Lisnizer N, Gatto A, and Soria G. 2004. Diving behavior and foraging areas of the Neotropic Cormorant at a marine colony in Patagonia, Argentina. *The Wilson Bulletin* 116:83-88.

98. Grémillet D, Kuntz G, Woakes AJ, Gilbert C, Robin J-P, Le Maho Y, and Butler PJ. 2005. Year-round recordings of behavioural and physiological parameters reveal the survival strategy of a poorly insulated diving endotherm during the Arctic winter. *Journal of Experimental Biology* 208:4231-4241.

99. Croxall J, Naito Y, Kato A, Rothery P, and Briggs D. 1991. Diving patterns and performance in the Antarctic blue‐eyed shag Phalacrocorax atriceps. *Journal of Zoology* 225:177-199.

100. Bevan R, Boyd I, Butler P, Reid K, Woakes A, and Croxall J. 1997. Heart rates and abdominal temperatures of free-ranging South Georgian shags, Phalacrocorax georgianus. *Journal of Experimental Biology* 200:661-675.

101. Wanless S, Harris M, and Morris J. 1992. Diving behaviour and diet of the blue-eyed shag at South Georgia. *Polar Biology* 12:713-719.

102. Sapoznikow A, and Quintana F. 2003. Foraging behavior and feeding locations of Imperial Cormorants and Rock Shags breeding sympatrically in Patagonia, Argentina. *Waterbirds* 26:184-191.

103. Quintana F. 1999. Diving behavior of Rock Shags at a Patagonian colony of Argentina. *Waterbirds*:466-471.

104. Wanless S, and Harris M. 1991. Diving patterns of full-grown and juvenile Rock Shags. *Condor*:44-48.

105. Tremblay Y, Cook TR, and Cherel Y. 2005. Time budget and diving behaviour of chick-rearing Crozet shags. *Canadian Journal of Zoology* 83:971-982.

106. Trayler K, Brothers D, Wooller R, and Potter I. 1989. Opportunistic foraging by three species of cormorants in an Australian estuary. *Journal of Zoology* 218:87-98.

107. Lea SE, Daley C, Boddington PJ, and Morison V. 1996. Diving patterns in shags and cormorants (Phalacrocorax): tests of an optimal breathing model. *Ibis* 138:391-398.

108. Ainley DG. 1990. *Seabirds of the Farallon Islands: ecology, dynamics, and structure of an upwelling-system community*: Stanford University Press.

109. Kato A, Naito Y, Watanuki Y, and Shaughnessy PD. 1996. Diving pattern and stomach temperatures of foraging king cormorants at subantarctic Macquarie Island. *The Condor* 98:844-848.

110. Kato A, Watanuki Y, Nishiumi I, Kuroki M, Shaughnessy P, and Naito Y. 2000. Variation in foraging and parental behavior of king cormorants. *The Auk* 117:718-730.

111. Cook T. 2008. The ecology of diving birds (Phalacrocorax spp.)(ecophysiological, behavioral and sexual responses to environment variability)PhD. Université de La Rochelle.

112. LaBastille A. 1974. Ecology and management of the Atitlán Grebe, Lake Atitlán, Guatemala. *Wildlife monographs* 37:3-66.

113. Huin N. 1994. Diving depths of white-chinned petrels. *The Condor* 96:1111-1113.

114. Myhrvold NP, Baldridge E, Chan B, Sivam D, Freeman DL, and Ernest S. 2015. An amniote life‐history database to perform comparative analyses with birds, mammals, and reptiles. *Ecology* 96:3109-3109.

115. Aguilar JS, Benvenuti S, Dall'Antonia L, McMinn-Grivé M, and Mayol-Serra J. 2003. Preliminary results on the foraging ecology of Balearic shearwaters (Puffinus mauretanicus) from bird-borne data loggers. *Scientia Marina* 67:129-134.

116. Watanuki Y, Kato A, Mori Y, and Naito Y. 1993. Diving performance of Adélie penguins in relation to food availability in fast sea-ice areas: comparison between years. *Journal of Animal Ecology*:634-646.

117. Watanuki Y, Miyamoto Y, and Kato A. 1999. Dive bouts and feeding sites of Adélie penguins rearing chicks in an area with fast sea-ice. *Waterbirds*:120-129.

118. Kato A, Yoshioka A, and Sato K. 2009. Foraging behavior of Adélie penguins during incubation period in Lützow-Holm Bay. *Polar Biology* 32:181-186.

119. Chappell MA, Shoemaker VH, Janes DN, Bucher TL, and Maloney SK. 1993. Diving behavior during foraging in breeding Adelie penguins. *Ecology* 74:1204-1215.

120. Culik B, Wilson R, and Bannasch R. 1994. Underwater swimming at low energetic cost by pygoscelid penguins. *Journal of Experimental Biology* 197:65-78.

121. Trivelpiece WZ, Bengtson JL, Trivelpiece SG, and Volkman NJ. 1986. Foraging behavior of Gentoo and Chinstrap penguins as determined by new radiotelemetry techniques. *The Auk*:777-781.

122. Mori Y. 1997. Dive bout organization in the chinstrap penguin at Seal Island, Antarctica. *Journal of Ethology* 15:9-15.

123. Luna-Jorquera G, Culik BM. 1999. Diving behaviour of Humboldt penguins Spheniscus humboldti in northern Chile*.* *Marine Ornithology* 27: 67-76

124. Mills K. 2000. Diving behaviour of two Galápagos Penguins Spheniscus mendiculus*.* *Marine Ornithology* 28:75-79

125. Zavalaga CB, et al. 2007. Diving behavior of blue-footed boobies Sula nebouxii in northern Peru in relation to sex, body size and prey type*.* *Marine Ecology Progress Series* 336:291-303

126. Weimerskirch H, et al. 2005. The three-dimensional flight of red-footed boobies: adaptations to foraging in a tropical environment? *Proceedings of the Royal Society of London B: Biological Sciences* 272(1558): 53-61

127. Ortega-Jiménez VM, et al. 2010. Takeoff flight performance and plumage wettability in Cassin’s auklet Ptychoramphus aleuticus, Xantus’s murrelet *Synthliboramphus hypoleucus* and Leach’s storm-petre*l Oceanodroma leucorhoa.* *Journal of Ornithology* 151(1):169-177

128. Hamilton CD, Golightly RT, Takekawa JY. 2005. Characteristics of diving in radio-marked Xantus's Murrelets*.* *Marine Ornithology* 33(2):155-159

129. Ropert-Coudert Y, Kato A. 2009. Diving activity of hoary-headed *(Poliocephalus poliocephalus)* and australasian little *(Tachybaptus novaehollandiae)* grebes*.* *Waterbirds* 32(1):157-161

130. Croll DA, et al. 1992. Foraging behavior and physiological adaptation for diving in thick-billed murres*.* *Ecology* 73(1): 344-35

131. Mori Y, et al. 2002. An application of optimal diving models to diving behaviour of Brünnich's guillemots*.* *Animal Behaviour* 64(5): 739-745

132. Falk K, et al. 2002. Foraging behaviour of thick-billed murres breeding in different sectors of the North Water polynya: an inter-colony comparison*.* *Marine Ecology Progress Series* 231:293-302

133. Falk K, et al. 2000. Time allocation and foraging behaviour of chick-rearing Brünnich's Guillemots *Uria lomvia* in high-arctic Greenland*.* *Ibis* 142(1): 82-92.

134. Somers MJ. 2000. Foraging behaviour of Cape clawless otters *(Aonyx capensis*) in a marine habitat*.* *Journal of Zoology* 252(4):473-480

135. Trillmich F, Kooyman GL, Majluf P. 1986. South American fur seals. Pages 153-167 in: Gentry RL, Kooyman GL, eds. *Fur seals: maternal strategies on land and at sea.* Princeton University Press, Princenton, NJ.

136. Mattlin R, Gales N, Costa D. 1998. Seasonal dive behaviour of lactating New Zealand fur seals *(Arctocephalus forsteri).* *Canadian Journal of Zoology* 76(2):350-360

137. Baylis AM, et al. 2005. The ontogeny of diving behaviour in New Zealand fur seal pups *(Arctocephalus forsteri).* *Canadian Journal of Zoology* 83(9):1149-1161

138. Page B, et al. 2005. Drift dives by male New Zealand fur seals *(Arctocephalus forsteri).* *Canadian Journal of Zoology* 83(2):293-300

139. Horning M, Trillmich F. 1997. Ontogeny of diving behaviour in the Galapagos fur seal*.* *Behaviour* 134(15):1211-1257

140. Kooyman GL, Trillmich F. 1986. Diving behavior of Galapagos fur seals. Pages 186-195 in: Gentry RL, Kooyman GL, eds. *Fur seals. Maternal strategies on land and at sea.* Princeton University Press, Princeton, NJ

141. Boyd I, Croxall J. 1992. Diving behaviour of lactating Antarctic fur seals*.* *Canadian Journal of Zoology* 70(5):919-928

142. Boyd IL, Reid K, Bevan RM. 1995. Swimming speed and allocation of time during the dive cycle in Antarctic fur seals*.* *Animal Behaviour* 50(3):769-784

143. McCafferty D, Boyd I, Taylor R. 1998. Diving behaviour of Antarctic fur seal *(Arctocephalus gazella)* pups*.* *Canadian Journal of Zoology* 76(3):513-520

144. Boyd I, McCafferty D, Walker T. 1997. Variation in foraging effort by lactating Antarctic fur seals: response to simulated increased foraging costs*.* *Behavioral Ecology and Sociobiology* 40(3):135-144

145. Lea MA, et al. 2002. Variability in the diving activity of Antarctic fur seals, *Arctocephalus gazella*, at Iles Kerguelen*.* *Polar Biology* 25(4):269-279

146. Robinson S, et al. 2003. The foraging ecology of two sympatric fur seal species*, Arctocephalus gazella* and *Arctocephalus tropicalis,* at Macquarie Island during the austral summer*.* *Marine and Freshwater Research* 53(7):1071-1082

147. Kooyman G, Davis R, Croxall J. 1986. Diving behavior of Antarctic fur seals*.* In: Gentry RL, Kooyman GL, eds. *Fur seals. Maternal strategies on land and at sea.* Princeton University Press, Princeton, NJ. 115-125

148. Costa DP, Goebel ME, Sterling JT. 2000. Foraging energetics and diving behavior of the Antarctic fur seal*, Arctocephalus gazella* at Cape Shirreff, Livingston Island. In: Davison W, Howard-Williams C, Broady P, eds. *Antarctic ecosystems: models for wider ecological understanding.* New Zealand Natural Sciences*,* Christchurch, Caxton press. 77-84

149. Goebel ME, et al. 2000. Foraging ranges and dive patterns in relation to bathymetry and time-of-day of Antarctic fur seals, Cape Shirreff, Livingston Island, Antarctica, In: Davison W, Howard-Williams C, Broady P, eds. *Antarctic ecosystems: models for wider ecological understanding.* New Zealand Natural Sciences*,* Christchurch, Caxton press. 47-50

150. Boyd I, Croxall J. 1996. Dive durations in pinnipeds and seabirds*.* *Canadian Journal of Zoology* 74(9):1696-1705

151. Francis J, Boness D, Ochoa-Acuña H. 1998. A protracted foraging and attendance cycle in female Juan Fernandez fur seals*.* *Marine Mammal Science* 14(3): 552-574

152. Arnould JP, Hindell MA. 2001. Dive behaviour, foraging locations, and maternal-attendance patterns of Australian fur seals *(Arctocephalus pusillus doriferus).* *Canadian Journal of Zoology* 79(1):35-48

153. Hindell MA, Pemberton D. 1997. Successful use of a translocation program to investigate diving behavior in a male Australian fur seal*, Arctocephalus pusillus doriferus*. *Marine Mammal Science* 13(2):219-228

154. Lander ME, Gulland FM, DeLong RL. 2000. Satellite tracking a rehabilitated Guadalupe fur seal *(Arctocephalus townsendi)*. *Aquatic Mammals* 26(2):137-142

155. Lea MA, Dubroca L. 2003. Fine-scale linkages between the diving behaviour of Antarctic fur seals and oceanographic features in the southern Indian Ocean*.* *ICES Journal of Marine Science*: *Journal du Conseil*, 60(5):990-1002

156. Georges JY, Tremblay Y, Guinet C. 2000. Seasonal diving behaviour in lactating subantarctic fur seals on Amsterdam Island*.* *Polar Biology* 23(1): 59-69

157. Cummings WC. 1985. Bryde’s whale *Balaenoptera edeni* Anderson, 1878. In: Ridgway SH, Harrison R, eds. *Handbook of marine mammals*. Academic press: London. 137-154

158. Nishiwaki M. 1950. On the body weight of whales*.* *Scientific Report Whales Research Institute* 4:184-209

159. Hobson R, Martin A. 1996. Behaviour and dive times of Arnoux's beaked whales, *Berardius arnuxii*, at narrow leads in fast ice*.* *Canadian Journal of Zoology* 74(2):388-393

160. Ponganis PJ, et al. 1992. Analysis of swim velocities during deep and shallow dives of two northern fur seals, *Callorhinus ursinus.* *Marine Mammal Science* 8(1):69-75

161. Sterling J, Ream RR. 2004. At-sea behavior of juvenile male northern fur seals (*Callorhinus ursinus*). *Canadian Journal of Zoology* 82(10):1621-1637

162. McIntyre IW, Campbell KL, MacArthur RA. 2002. Body oxygen stores, aerobic dive limits and diving behaviour of the star-nosed mole *(Condylura cristata*) and comparisons with non-aquatic talpids*.* *Journal of Experimental Biology* 205(1): 45-54

163. Martin A, Smith T, Cox O. 1998. Dive form and function in belugas *Delphinapterus leucas* of the eastern Canadian High Arctic*.* *Polar Biology* 20(3):218-228

164. Ridgway S, et al. 1984. Diving and blood oxygen in the white whale*.* *Canadian Journal of Zoology* 62(11):2349-2351

165. Shaffer SA, et al. 1997. Diving and swimming performance of white whales*, Delphinapterus leucas*: an assessment of plasma lactate and blood gas levels and respiratory rates*.* *Journal of Experimental Biology* 200(24): 3091-3099

166. Lerczak JA, Shelden KE, Hobbs RC. 2000. Application of suction-cup-attached VHF transmitters to the study of beluga, *Delphinapterus leucas*, surfacing behavior in Cook Inlet, Alaska. *Marine Fisheries Review* 62(3): 99-111

167. Chilvers BL, et al. 2004. Diving behaviour of dugongs, *Dugong dugon*. *Journal of Experimental Marine Biology and Ecology* 304(2):203-224

168. Anderson P, Birtles A. 1978. Behaviour and Ecology of the Dugong*, Dugong Dugon* (Sirenia): Observations in Shoalwater and Cleveland Bays, Queensland. *Wildlife Research* 5(1):1-23

169. Yeates LC, Williams TM, Fink TL. 2007. Diving and foraging energetics of the smallest marine mammal, the sea otter *(Enhydra lutris).* *Journal of Experimental Biology* 210(11):1960-1970

170. Gjertz I, et al. 2000. Movements and diving of adult ringed seals (*Phoca hispida*) in Svalbard*.* *Polar Biology* 23(9): 651-656

171. Krafft BA, et al. 2000. Diving behaviour of lactating bearded seals (*Erignathus barbatus*) in the Svalbard area*.* *Canadian Journal of Zoology* 78(8):1408-1418

172. Gjertz I, et al. 2000. Movements and diving of bearded seal (*Erignathus barbatus*) mothers and pups during lactation and post-weaning. *Polar Biology* 23(8):559-566

173. Winn HE, et al. 1995. Dive patterns of tagged right whales in the Great South Channel*.* *Continental Shelf Research* 15(4): 593-611

174. Merrick RL, Loughlin TR. 1997. Foraging behavior of adult female and young-of-the-year Steller sea lions in Alaskan waters. *Canadian Journal of Zoology* 75(5):776-786

175. Goulet AM, Hammill MO, Barrette C. 2001. Movements and diving of grey seal females (*Halichoerus grypus*) in the Gulf of St. Lawrence, Canada*.* *Polar Biology* 24(6):432-439

176. Lydersen C, Hammill MO, Kovacs KM. 1994. Activity of lactating ice-breeding grey seals, *Halichoerus grypus*, from the Gulf of St Lawrence, Canada*.* *Animal Behaviour* 48(6):1417-1425

177. Beck CA, et al. 2003. Sex differences in the diving behaviour of a size-dimorphic capital breeder: the grey seal*.* *Animal Behaviour* 66(4):777-789

178. Austin D, et al. 2006. Stomach temperature telemetry reveals temporal patterns of foraging success in a free-ranging marine mammal*.* *Journal of Animal Ecology* 75(2): 408-420

179. Beck CA, et al. 2003. Sex differences in diving at multiple temporal scales in a size-dimorphic capital breeder*.* *Journal of Animal Ecology* 72(6):979-993

180. Lidgard DC, et al. 2003. Diving behaviour during the breeding season in the terrestrially breeding male grey seal: implications for alternative mating tactics*.* *Canadian Journal of Zoology* 81(6): 1025-1033

181. Kuhn CE, et al. 2006. Diving physiology and winter foraging behavior of a juvenile leopard seal (*Hydrurga leptonyx*)*.* *Polar Biology* 29(4):303-307

182. Hooker SK, Baird RW. 1999. Deep–diving behaviour of the northern bottlenose whale, Hyperoodon ampullatus (Cetacea: Ziphiidae). *Proceedings of the Royal Society of London B: Biological Sciences* 266(1420):671-676

183. Nowak RM. 1999. Order Cetacea*.* In: Nowak RM, ed. *Walker's Mammals of the World*. JHU Press: Baltimore and London. 896-981

184. Barlow J, et al. 1997. A report of cetacean acoustic detection and dive interval studies (CADDIS) conducted in the southern Gulf of California. *NOAA Technical Memorandum, NOAA-TM-NMFS-SWFSC-250*

185. Mate BR, et al. 1994. Movements and dive behavior of a satellite-monitored Atlantic white-sided dolphin (*Lagenorhynchus acutus*) in the Gulf of Maine*.* *Marine Mammal Science* 10(1):116-121

186. Bodley K, van Polanen Petel T, Gales N. 2005. Immobilisation of free-living Weddell seals *Leptonychotes weddellii* using midazolam and isoflurane*.* *Polar Biology* 28(8):631-636

187. Burns JM. 1999. The development of diving behavior in juvenile Weddell seals: pushing physiological limits in order to survive. *Canadian Journal of Zoology* 77(5):737-747

188. Castellini MA, Davis RW, Kooyman GL. 1988. Blood chemistry regulation during repetitive diving in Weddell seals. *Physiological Zoology* 61:379-386.

189. Peixun C. 1989. Baiji (*Lipotes vexillifer*) Miller, 1918*.* In: Ridgway SH, Harrison R, eds. *Handbook of marine mammals*. Academic press: London. 25-43

190. Bengtson JL, Stewart BS. 1992. Diving and haulout behavior of crabeater seals in the Weddell Sea, Antarctica, during March 1986*.* *Polar Biology* 12(6-7):635-644

191. Dolphin WF. 1987. Ventilation and dive patterns of humpback whales, *Megaptera novaeangliae,* on their Alaskan feeding grounds. *Canadian Journal of Zoology* 65(1):83-90

192. Boeuf BJL, et al. 1988. Continuous, deep diving in female northern elephant seals, *Mirounga angustirostris.* *Canadian Journal of Zoology* 66(2):446-458

193. Naito Y, et al. 1989. Long-term diving records of an adult female northern elephant seal. *Antarctic Records* 33(1):1-9

194. Boeuf BJL, et al. 1989. Prolonged, continuous, deep diving by northern elephant seals*.* *Canadian Journal of Zoology* 67(10):2514-2519

195. Boeuf BJL, et al. 2000. Respiration and heart rate at the surface between dives in northern elephant seals*.* *Journal of Experimental Biology* 203(21):3265-3274

196. Davis RW, et al. 2001. Three-dimensional movements and swimming activity of a northern elephant seal*.* *Comparative Biochemistry and Physiology Part A: Molecular & Integrative Physiology* 129(4): 759-770

197. Webb P, et al. 1998. Effects of buoyancy on the diving behavior of northern elephant seals. *Journal of Experimental Biology* 201(16):2349-2358

198. Boeuf BJL, et al. 1996. Diving behavior of juvenile northern elephant seals. *Canadian Journal of Zoology* 74(9):1632-1644

199. Slip DJ, Hindell MA, and Burton HR. 1994. Diving behavior of southern elephant seals from Macquarie Island: an overview. *Elephant seals: population ecology, behavior, and physiology University of California Press, Berkeley*:253-270.

200. Hindell M, Slip D, Burton H. 1991. The diving behavior of adult male and female southern elephant seals, *Mirounga leonina* (Pinnipedia, Phocidae). *Australian Journal of Zoology* 39(5): 595-619

201. Hindell MA, et al. 2000. Metabolic limits on dive duration and swimming speed in the southern elephant seal *Mirounga leonina.* *Physiological and Biochemical Zoology* 73(6):790-798

202. Kiraç CO, et al. 2002. Observations on diving beahviour of free-ranging Mediterranean monk seals *Monachus monachus* on Turkish coasts*.* *The Monachus Guardian* 5(1):37-42

203. Köhler D. 1991. Notes on the diving behaviour of the water shrew*, Neomys fodiens* (Mammalia, Soricidae). *Zoologischer Anzeiger* 227(3-4):218-228

204. Costa DP, Gales NJ. 2003. Energetics of a benthic diver: seasonal foraging ecology of the Australian sea lion, *Neophoca cinerea.* *Ecological Monographs* 73(1):27-43

205. Fowler SL, et al. 2006. Ontogeny of diving behaviour in the Australian sea lion: trials of adolescence in a late bloomer*.* *Journal of Animal Ecology* 75(2):358-367

206. Akamatsu T, et al. 2000. A method for individual identification of echolocation signals in free-ranging finless porpoises carrying data loggers*.* *The Journal of the Acoustical Society of America* 108(3):1353-1356

207. Born EW, et al. 2005. Homing behaviour in an Atlantic walrus (*Odobenus rosmarus rosmarus*)*.* Aquatic Mammals 31(1):23-33

208. Southwell C. 2005. Diving behaviour of two Ross seals off east Antarctica*.* *Wildlife Research* 32(1):63-65

209. MacArthur RA, Weseen GL, Campbell KL. 2003. Diving experience and the aerobic dive capacity of muskrats: does training produce a better diver? *Journal of Experimental Biology*, 206(7):1153-1161

210. Stacey PJ, Hvenegaard GT. 2002. Habitat use and behaviour of Irrawaddy dolphins (*Orcaella brevirostris*) in the Mekong River of Laos. *Aquatic Mammals* 28(1):1-13

211. Baird RW, Hanson MB, Dill LM. 2005. Factors influencing the diving behaviour of fish-eating killer whales: sex differences and diel and interannual variation in diving rates*.* *Canadian Journal of Zoology* 83(2):257-267

212. Bethge P, et al. 2003. Diving behaviour, dive cycles and aerobic dive limit in the platypus *Ornithorhynchus anatinus.* *Comparative Biochemistry and Physiology Part A: Molecular & Integrative Physiology* 136(4):799-809

213. Otley HM, Munks SA, Hindell MA. 2000. Activity patterns, movements and burrows of platypuses (*Ornithorhynchus anatinus*) in a sub-alpine Tasmanian lake*.* *Australian Journal of Zoology* 48(6):701-713

214. Thompson D, et al. 1998. Foraging behaviour and diet of lactating female southern sea lions (*Otaria flavescens*) in the Falkland Islands*.* *Journal of Zoology* 246(2):135-146

215. Werner R, Campagna C. 1995. Diving behaviour of lactating southern sea lions (*Otaria flavescens*) in Patagonia. *Canadian Journal of Zoology* 73(11):1975-1982.

216. Folkow LP, Nordøy ES, Blix AS. 2004. Distribution and diving behaviour of harp seals (*Pagophilus groenlandicus*) from the Greenland Sea stock*.* *Polar Biology* 27(5): 281-298

217. Lydersen C, Kovacs KM. 1993. Diving behaviour of lactating harp seal, *Phoca groenlandica,* females from the Gulf of St Lawrence, Canada. *Animal Behaviour* 46(6):1213-1221

218. Kelly BP, Wartzok D. 1996. Ringed seal diving behavior in the breeding season*.* *Canadian Journal of Zoology* 74(8):1547-1555

219. Hyvärinen H, Hämäläinen E, Kunnasranta M. 1995. Diving behavior of the Saimaa ringed seal (*Phoca hispida saimensis* Nordq.)*.* *Marine Mammal Science* 11(3):324-334

220. Watanabe Y, et al. 2004. Foraging tactics of Baikal seals differ between day and night*.* *Marine Ecology Progress Series* 279: 283-289

221. Stewart BS, et al. 1996. Seasonal movements and dive patterns of juvenile Baikal seals, *Phoca sibirica.* *Marine Mammal Science* 12(4):528-542

222. Eguchi T, Harvey JT. 2005. Diving behavior of the Pacific harbor seal (*Phoca vitulina richardii*) in Monterey Bay, California. *Marine Mammal Science* 21(2):283-295

223. Bowen W, Boness D, Iverson SJ. 1999. Diving behaviour of lactating harbour seals and their pups during maternal foraging trips*.* *Canadian Journal of Zoology* 77(6):978-988

224. Boness D, Bowen W, Oftedall O. 1994. Evidence of a maternal foraging cycle resembling that of otariid seals in a small phocid, the harbor seal*.* *Behavioral Ecology and Sociobiology* 34(2):95-104

225. Stewart BS, et al. 1989. Harbor seal tracking and telemetry by satellite*.* *Marine Mammal Science* 5(4):361-375

226. Jørgensen C, et al. 2001. Diving development in nursing harbour seal pups*.* *Journal of Experimental Biology* 204(22):3993-4004

227. Bowen W, et al. 2002. Prey-dependent foraging tactics and prey profitability in a marine mammal*.* *Marine Ecology Progress Series* 244:235-245

228. Gales N, Mattlin R. 1997. Summer diving behaviour of lactating New Zealand sea lions, Phocarctos hookeri. *Canadian Journal of Zoology* 75(10):1695-1706

229. Crocker D, Gales N, Costa D. 2001. Swimming speed and foraging strategies of New Zealand sea lions (*Phocarctos hookeri*)*.* *Journal of Zoology.* 254(02):267-277

230. Costa DP, Gales NJ. 2000. Foraging energetics and diving behavior of lactating New Zealand sea lions, *Phocarctos hookeri.* *Journal of Experimental Biology* 203(23):3655-3665

231. Chilvers B, et al. 2006. Diving to extremes: are New Zealand sea lions (*Phocarctos hookeri*) pushing their limits in a marginal habitat? *Journal of Zoology* 269(2):233-240

232. Otani S, et al. 1998. Diving behavior and performance of harbor porpoises, *Phocoena phocoena,* in Funka Bay, Hokkaido, Japan*.* *Marine Mammal Science* 14(2):209-220

233. Otani S, et al. 2000. Diving behavior and swimming speed of a free-ranging harbor porpoise, *Phocoena phocoena.* *Marine Mammal Science* 16(4):811-814

234. Otani S, et al. 2001. Oxygen consumption and swim speed of the harbor porpoise *Phocoena phocoena.* *Fisheries Science* 67(5):894-898

235. Westgate AJ, et al. 1995. Diving behaviour of harbour porpoises*, Phocoena phocoena.* *Canadian Journal of Fisheries and Aquatic Sciences* 52(5):1064-1073

236. Watson A, Gaskin D. 1983. Observations on the ventilation cycle of the harbour porpoise *Phocoena phocoena* (L.) in coastal waters of the Bay of Fundy*.* *Canadian Journal of Zoology* 61(1):126-132

237. Hanson MB, Baird RW. 1998. Dall's porpoise reactions to tagging attempts using a remotely-deployed suction-cup tag*.* *Marine Technology Society Journal* 32(2):18-23

238. Amano M, Yoshioka M. 2003. Sperm whale diving behavior monitored using a suction-cup-attached TDR tag*.* *Marine Ecology Progress Series* 258:291-295

239. Watkins WA, et al. 1993. Sperm whales tagged with transponders and tracked underwater by sonar*.* *Marine Mammal Science* 9(1):55-67

240. Edwards HH, Schnell GD. 2001. Body length, swimming speed, dive duration, and coloration of the dolphin *Sotalia fluviatilis* (Tucuxi) in Nicaragua*.* *Caribbean Journal of Science* 37(3/4):271-271

241. Davis RW, et al. 1996. Diving behavior and at-sea movements of an Atlantic spotted dolphin in the Gulf of Mexico*.* *Marine Mammal Science* 12(4):569-581

242. Dyck M, Romberg S. 2007. Observations of a wild polar bear (*Ursus maritimus*) successfully fishing Arctic charr (*Salvelinus alpinus*) and Fourhorn sculpin (*Myoxocephalus quadricornis*). *Polar Biology* 30(12):1625-1628

243. Feldkamp SD, DeLong RL, Antonelis GA. 1989. Diving patterns of California sea lions, *Zalophus californianus.* *Canadian Journal of Zoology* 67(4):872-883

1. Mean temperature of all bird species in compilation [34] [↑](#endnote-ref-1)
2. Mean temperature of bird species in genus [34] [↑](#endnote-ref-2)
3. Mean temperature of all mammal species in compilation [34] [↑](#endnote-ref-3)
4. Mean temperature of mammal species in genus [34] [↑](#endnote-ref-4)
